# Portfolio optimization for seed selection in diverse weather scenarios

**DOI:** 10.1371/journal.pone.0184198

**Published:** 2017-09-01

**Authors:** Oskar Marko, Sanja Brdar, Marko Panić, Isidora Šašić, Danica Despotović, Milivoje Knežević, Vladimir Crnojević

**Affiliations:** 1 BioSense Institute, University of Novi Sad, Novi Sad, Serbia; 2 Faculty of Technical Sciences, University of Novi Sad, Novi Sad, Serbia; College of Agricultural Sciences, UNITED STATES

## Abstract

The aim of this work was to develop a method for selection of optimal soybean varieties for the American Midwest using data analytics. We extracted the knowledge about 174 varieties from the dataset, which contained information about weather, soil, yield and regional statistical parameters. Next, we predicted the yield of each variety in each of 6,490 observed subregions of the Midwest. Furthermore, yield was predicted for all the possible weather scenarios approximated by 15 historical weather instances contained in the dataset. Using predicted yields and covariance between varieties through different weather scenarios, we performed portfolio optimisation. In this way, for each subregion, we obtained a selection of varieties, that proved superior to others in terms of the amount and stability of yield. According to the rules of Syngenta Crop Challenge, for which this research was conducted, we aggregated the results across all subregions and selected up to five soybean varieties that should be distributed across the network of seed retailers. The work presented in this paper was the winning solution for Syngenta Crop Challenge 2017.

## Introduction

As the world’s demand for food grows [1], a huge pressure is being put on agriculture to produce more. In the light of recent studies that show stagnation and decline in yield improvement [2], as well as estimates that yield is not improving fast enough to meet the demands forecasted for 2050 [3], producing enough food has become a global challenge.

Agricultural performance is very sensitive to a wide range of factors such as weather, soil, land management and their complex interplay. Research on a global scale implies that climate variation explains around one third of worldwide crop yield variability [4]. On a local scale, weather effects on the crops can be further specified and utilised for predictive modelling. For example, temperature and precipitation, along with evapotranspiration of plants during the growing season can be used for highly accurate yield prediction [5, 6]. In-depth study of temperature throughout plant life cycle can unveil stages of development that are particularly susceptible to temperature extremes [7] or can identify how different weather patterns influence yield and leverage them for proposing weather and site specific recommendations [8]. Even aggregated features such as cumulative precipitation in a year and annual mean temperature [8] or cumulative rainfall and solar radiation, or seasonal maximal, average and minimal temperatures provide valuable information on climate-crop interactions [9].

Another important aspect of agricultural production is the quality of soil. It was shown that soil parameters, such as pH value [10–13], texture [10, 11], cation exchange capacity [10, 13], soil type [10, 12] and content of phosphorus, calcium, magnesium and potasium [13] can be valuable features for yield prediction. The only problem is that these parameters are hard to acquire. Soil examination involves field sampling and laboratory analysis, which are time-consuming and labourous tasks and may include additional costs that farmers are often not ready to pay. For that reason, sometimes, as in [11], studies rely on data provided by farmers according to a predifined method, such as RASTA (Rapid Soil and Terrain Assessment, available online at http://www.open-aeps.org/RASTA.pdf) [14]. This method aids farmers in assessing soil type, texture and chemical composition on their own and although the acquired data may not be especially accurate, using it in conjunction with weather parameters can bring significant improvement to the model.

Different climates and soil conditions require appropriate seed varieties and thus smart seed selection shows a lot of promise in boosting crop production. Agricultural systems are made resilient in conditions of weather-induced stress by providing access to diverse seed varieties and applying advanced seed selection strategies [15]. Farmers are generally willing to pay for seed-related information [16]. Although willingness to do so is limited by their income, seed information indeed has a market that could be utilised by agricultural extension services or even industry. Years of observations and experiments produced rich data collections and opened the avenue for data analytics in agriculture, where knowledge and information have a central role.

Choosing the right variety or a portfolio of varieties for sowing is an optimisation problem. There are no varieties that perform well in all conditions. Some are more suitable for wet, others for dry seasons. Some prefer sandy and others clay soils. Nevertheless, it is usually the ones more resilient to weather conditions and diseases, that have average yields, in contrast to high-yielding varieties that are more likely to fail when the growing conditions are not perfect. Trade-off between high yield and low risk is the basis of the optimisation problem that can be mathematically defined and solved using modern portfolio theory set by Harry Markowitz in 1952 [17]. Originally, this theory was developed for solving the problem of optimal choice of assets in which investments should be placed. It is used for minimisation of risk for a desired return, or likewise, for maximisation of return for a desired level of risk. Since 1950s, there have been problems from many other areas in which this theory was applied, such as the problem of activation of power stations under ecological constraints [18], water supply projection under weather and political risks [19], coastal ecosystem management under environmental and military constraints [20], and optimal forestation in the context of wood production, carbon sequestration and economic valorisation [21]. Agriculture is no exception. Portfolio optimisation approach has been applied in crop selection for achieving the optimal trade-off between profit and environmental footprint [22] and in soybean [10], rice [23] and wheat [24] variety selection for risk minimisation and yield maximisation.

### Syngenta crop challenge

The aim of Syngenta Crop Challenge [25] is to promote the use of data analytics for solving problems in agriculture. While the 2016 challenge was directed towards designing the optimal strategy for seed selection from the farmer’s point of view, the 2017 challenge focused on seed retailers. The goal was to select up to five soybean varieties with the biggest potential yield, which are consequently the most likely to be sold. The retailers would then use this information to prepare for the forthcoming season by acquiring the right selection of seeds and in the right amount. Shortage of seeds meant that the profit would be lower than what they could have achieved, whereas too many seeds would leave them with a stockpile of commodities they cannot sell.

## Materials and methods

The first step towards selection of varieties was preprocessing of data, where feature selection and filling the gaps in data took place. The next was yield prediction, while in the last step risk was counted in. The amount and deviation of yield were the only decision criteria for seed selection. In the last step, results were aggregated across all of the 6,490 subregions and up to 5 varieties were selected with contributions of at least 10%, according to the Challenge rules.

### Data and preprocessing

**Fig 1:**
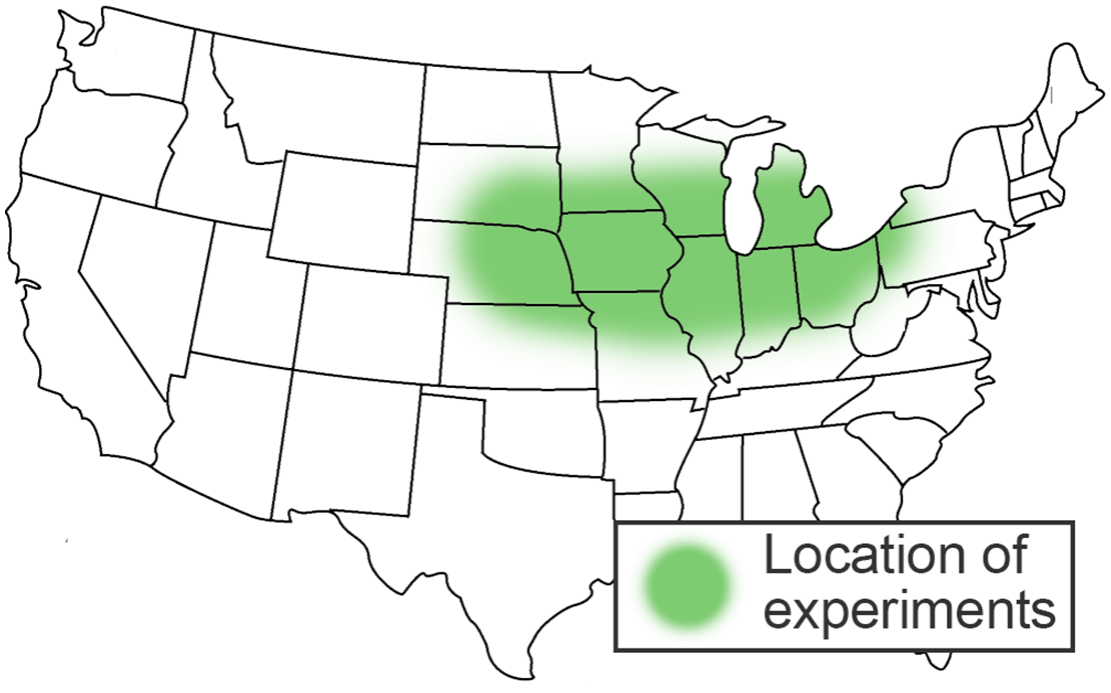
Region of the United States where data was acquired.

We had Experiment and Region dataset at our disposal. Experiment dataset represented the knowledge about performance of 174 soybean varieties under different weather and soil conditions and it was intended for model training and testing. It consisted of 82,036 observations made between 2009 and 2015 at 205 farms in the Midwestern United States (Fig 1).

The second one was Region dataset. It included the information about weather and soil conditions for years ranging from 2001 to 2015, in the region where proposed varieties would be planted. The growing region consisted of 6,490 subregions of size 36 mi^2^. Combined, they formed a territory roughly the size of France allowing for drastic weather variability. In this part of the world, extremely hot summers and harsh winters happen frequently. Rain is also common, especially in the eastern part of the Midwest. Features, along with their explanations, are shown in Table 1. All of them were included in Experiment dataset, while region dataset did not include information about yield, check yield, yield difference, soil class and planting date.

**Table 1:** List of features and their explanations.

Feature	Explanation
Yield	Amount of grain per unit of land
Check yield	Commercial variety’s yield used as performance benchmark
Yield difference	Difference between yield and check yield
Year	Year in which the variety was planted
Planting date	Planting date
Geographic coordinates	GPS latitude and longitude of the farm or region
Temperature	Sum of daily temperatures in *C*° in growing season
Precipitation	Sum of daily precipitations in *mm* in growing season
Solar radiation	Sum of daily solar radiation in *W*/*m*^2^ in growing season
CEC	Cation exchange capacity
pH	log of H+ concentration in soil
Organic matter	Percentage of soil made up of organic matter
Soil class	Soil class category
Clay	Percentage of clay in soil
Silt	Percentage of silt in soil
Sand	Percentage of sand in soil
Area	Probability of growing soybeans in the subregion
PI	Soil productivity index

Features available in the Experiment dataset are listed in the left column, while their explanations are located on the right.

Growing season in which weather measurements were aggregated was defined as the period between 1st April and 31st October. Both Experiment and Region datasets contained soil productivity index (PI) which referred to the potential of planted crops to have good yield on that particular soil. According to PI, soil was ranked using numerical values ranging from 0 to 19, with 0 denoting the least productive and 19 denoting the most productive soil type [26]. This measure was independent of landscape and geographic position, as it incorporated only soil taxonomy information. Weather and soil data were obtained from European Centre of Medium Range Weather Forecasts (ECMWF) and Soilgrids website [27], respectively, while the source for *area* parameter was USDA-NASS [28]. Soybeans are generally tolerant of poor soil, but grow best in well-drained soil rich in organic matter with pH around 6.5 [29].

The amount of data for each variety was unequal. Some varieties and farms were more present in the Experiment dataset and others less. There were missing observations for planting date and PI in Experiment dataset. Due to the absence of planting date and soil class in the region dataset, these features were not used in the analysis, while the missing values of PI were substituted with the mean of all the existing PIs.

**Fig 2:**
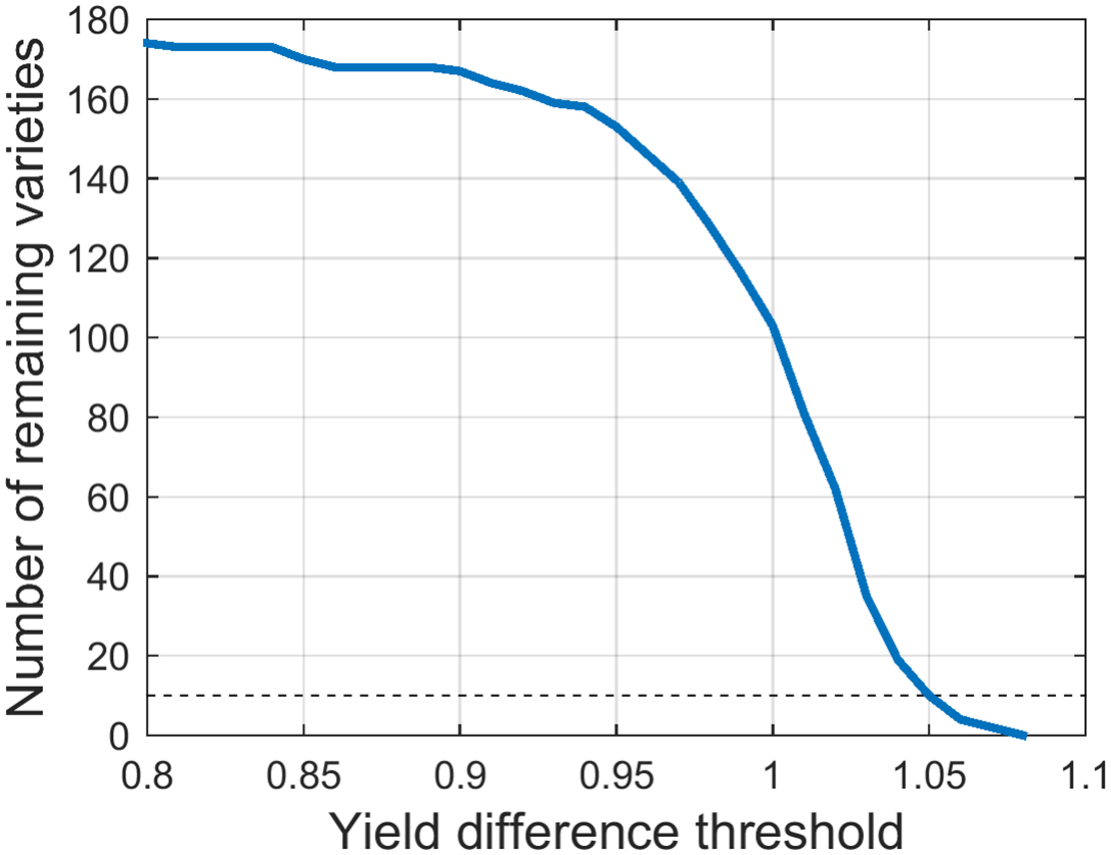
Elimination of varieties using yield difference threshold.

Instead of using absolute yield difference, we used the relative measure of it, i.e. the ratio between yield and check yield. It indicated whether the commercial variety was outperformed or not and by how much. Varieties that constantly had worse yield than commercial ones were filtered out. Optimal yield difference threshold (YDT) was obtained through brute force optimisation. YDT was varied between 0.8, where no varieties were eliminated and 1.05, where only 10 varieties left (Fig 2). Filtering out more varieties would suppress diversity in the final selection. The objective function for YDT optimisation is explained in more detail in a following section.

There were samples with the same location, planting date and variety, but with different yield which would cause ambiguity in the learning process. Such observations were merged into one observation with the yield equal to the mean yield of all of them. In this way, final number of samples fell from 82,036 to 19,432. Further insight into the dataset is given in Supporting information. Histograms of features and yield statistics of the preprocessed dataset are given in S1–16 and S17–19 Figs, respectively.

### Yield prediction

In order to select varieties according to the first criterion, i.e. the amount of yield, we needed to have a reliable prediction of their yields, for what we needed a model. The model input consisted of features shown in Table 2.

**Table 2:** Features that were considered.

Variety	CEC	Temperature
Year	Organic matter	Precipitation
GPS latitude	Clay	Solar radiance
GPS longitude	Silt	Area
pH	Sand	PI

List of features considered in the analysis, after removal of the ones that were not present in the Region dataset (check yield, yield difference, planting date and soil class).

Models were constructed using some of today’s most popular methods of machine learning, namely: multiple linear regression (MLR), random forest (RF), support vector machine (SVM), k-nearest neighbours (k-NN), artificial neural network (ANN) and weighted histograms regression (WHR).

MLR attempts to model the relationships within the data with linear equations. Consequently, any non-linear relationships are not likely to be successfully represented in the model.

RF is an ensemble learning method based on training a large number of decision trees. It is robust to outliers and does not have issues with overfitting [30]. The size of the dataset is rarely an issue, as the construction of trees does not require much time. Also, passing through trees is very fast as the computer essentially needs to go through a number of simple “if” statements. Also, RF can handle non-linear data and categorical features, e.g. variety’s name. For the yield prediction problem, a total of 100 trees were grown, with 5 features considered in each one.

Another method used for building the model was SVM [31]. Although primarily developed for solving classification problems, its regression variant was developed soon after [32]. We used MATLAB [33] implementation of the algorithm, based on *libsvm* package [34]. The problem with SVM is that it does not perform well in unbalanced datasets [35], such as this one where there was unequal amount of data for different varieties.

Although one of the less complex methods, k-NN [36] is used very often. Due to its simplicity, it performs well with large datasets. The main disadvantage of k-NN is its sensitivity to the “curse of dimensionality”, which happens when dimensionality of feature space is inappropriately high for the insufficient amount of data [37].

Designed as a concept analogous to the structure of brain, ANNs [38] are especially successful in resolving problems with complex, non-linear relations. When large datasets are available for training, neural networks can learn various patterns and generalise well [39]. We used a generalised regression neural network with 2 hidden layers, each having 100 neurons. The issue with ANN is that it often finds locally optimal weights of the neurons, instead of the global ones [40].

The last method that was examined, WHR, was designed as a part of the Syngenta Crop Challenge 2016 [10]. When yield is predicted for a certain variety at a test farm, histogram is made out of yields at all the training farms where this variety was planted. Furthermore, the histogram entries are weighted according to the similarity of features between the test and the training farms.

Standard validation methods such as leave-one-out or 10-fold cross-validation could not be used. The problem with them is that they allow training and test samples to be drawn from the same year and, because they grew under the same weather conditions, samples from the same year are very correlated in terms of yield. Results obtained in such way are too optimistic and do not correspond to the real accuracy of the model, which is somewhat lower. For this reason, we validated the results by taking one year’s data for testing and other years’ data for training. The splitting process was repeated until all of the 7 years were taken as the test years. In the end, results for all of the test/training years combinations were averaged. This “leave-one-year-out” better represented the real-world situation in which we do not know next year’s yields, but only the historical ones. We compared the results of different regression algorithms using following objective measures:

1. Root-mean-square error (RMSE)
  $$\begin{array}{c} RMSE=\sqrt{\frac{1}{N}\sum_{i=1}^{N}{\left(y_{i}-{\hat{y}}_{i}\right)}^{2}} \end{array}$$
2. Mean absolute error (MAE)
  $$\begin{array}{c} MAE=\frac{1}{N}\sum_{i=1}^{N}\left|y_{i}-{\hat{y}}_{i}\right| \end{array}$$
3. Pearson correlation coefficient (*r*)
  $$\begin{array}{c} r\left(Y,\hat{Y}\right)=\frac{cov(Y,\hat{Y})}{\sigma_{Y}\sigma_{\hat{Y}}}=\frac{E[\left(Y-\mu_{Y}\right)\left(\hat{Y}-\mu_{\hat{Y}}\right)]}{\sigma_{Y}\sigma_{\hat{Y}}} \end{array}$$
4. Spearman’s rank correlation coefficient (*ρ*)
  $$\begin{array}{c} \rho \left(Y,\hat{Y}\right)=r\left(rg\left(Y\right),rg\left(\hat{Y}\right)\right)=1-\frac{6\sum_{i=1}^{N}d_{i}}{N(N^{2}-1)} \end{array}$$

where *y* and $\hat{y}$ denote real and predicted values of a sample (real and predicted yield in this case), *N* denotes the total number of samples, *Y* and $\hat{Y}$ are random variables associated to real and predicted sample values, *μ* and *σ* denote the mean value and standard deviation of a random variable, *rg* converts a vector of values into a vector of ranks and *d*_*i*_ denotes the difference between ranks of predicted and real values at the position *i*. RMSE is sometimes a better measure of error than MAE, since it takes the square of error and hence penalises big outliers. Pearson’s correlation indicates how much does the dependence between predicted and measured yield deviate from linear. Spearman’s on the other hand indicates how much does the dependence between them deviates from monotonic, i.e. how well do their ranking orders correspond to one another. All of the regression algorithms mentioned above were implemented using MATLAB. We chose the machine learning method with least error and highest correlation coefficient for yield prediction and proceeded to the next stage.

### Global sensitivity and uncertainty analyses

The main problem with modern machine learning techniques is that they are essentially black box models. This means that rather than explaining phenomena, they only give outputs for sets of inputs. While linear regression, for example, allows us to easily comprehend the relationship between input and output parameters, advanced machine learning models are very difficult to analyse, due to a high level of non-linearity inside of them. Thus, the influence of different input parameters on output and their interaction between one another are quantified indirectly, through global sensitivity and uncertainty analyses. In particular, uncertainty analysis explores the possible values of outputs under the uncertainty of input factors. Probability density functions (PDFs) are assigned to inputs and the output value is calculated for a number of points from the input space, sampled following a Monte Carlo procedure. In this paper, we used uniform PDFs bounded by minimum and maximum values of features to simulate their uncertainty. After performing Monte Carlo experiments, a distribution of output values is generated, which reveals details about the possible outcomes of yield prediction. Whereas uncertainty analysis gives an estimate of the scope of the output, sensitivity analysis gives a more detailed insight into the relationship between individual input factors and output, as well as the interdependencies between the input factors. The two most popular mehtods for conducting both analyses were developed by Morris [41] and Sobol’ [42]. Morris method [41] is based on elementary effects analysis. It involves observation of the change in output due to variation in input factors, one at a time. Since the input factors generally have a different effect on the output in different points of the input space, elementary effects are calculated in a number of points, sampled either as vertices of a Latin hypercube or following a random algorithm [43]. The direct influence of an input factor on output is expressed as the mean absolute value of the elementary effect (μ*) [44], while its standard deviation (*σ*) indicates the level of interaction with other factors [41]. Although Morris method is performed in various locations of the input space, it is a one-at-a-time method and cannot be considered truly global. Sobol’ analysis, on the other hand, is a variance-based technique, which means that it decomposes the variance of the output into a sum of individual input variances and variances of their combinations [42]. Total influence (total sensitivity index) of the i-th factor (*S*_*T*_*i*__) is then calculated as [45]:

$$\begin{array}{c} S_{T_{i}}=S_{i}+S_{I_{i}}, \end{array}$$

where *S*_*i*_ and *S*_*I*_*i*__ are first-order sensitivity index and interaction index, respectively. Their interpretation is somewhat similar to μ* and *σ* from Morris method. First-order sensitivity index gives a measure of the direct influence a factor has on the output, while interaction index gives a measure of sensitivity of output to this factor combined with other ones. However, unlike the Morris method which gives a qualitative sensitivity estimates, Sobol’ method gives a quantitative measure of sensitivity. Furthermore, because of the in-depth study of mutual interactions between factors, it is indeed considered global. The only problem is that Sobol’ method is computationally very expensive. As all factor combinations need to be calculated, its complexity grows exponentially with the number of factors. Nevertheless, there are efficient algorithms which give robust estimates of sensitivity indices with reduced complexity. Modified Sobol’ method, introduced by Saltelli [46] requires only *M*(2*k* + 2) simulations, where *k* is the number of factors and *M* is usually a value between 500 and 1000 [43]. It is capable of calculating first, second, total and *k* − 2-nd index at once. However, global sensitivity analysis can still be time-consuming. For that reason, Morris method is usually used for screening. It is capable of identifying the most important factors in only *r*(*k* + 1) simulations, where *k* is the number of factors and r an integer between 4 and 10 [43]. After screening of the factors, variance-based analysis is performed on the most important ones to give a more precise estimate of their sensitivity [43, 47–50].

### Weather scenarios

Throughout the years, soil properties change considerably slower than weather conditions, which are different from one year to another. We therefore took weather as the main source of risk in soybean production. Other sources of risk include occurrence of plant diseases or animal pests, farmer’s budget for fertilisation, efficiency of agricultural machinery etc., but this aspect was not considered in the Challenge. Weather conditions can be understood as realisations of climate, which is the random variable in this case. We refer to weather conditions in one year, i.e. one realisation of climate variable, as one possible *weather scenario*. Next, we make two assumptions.

1. There are no other weather scenarios, but the 15 present in the region dataset.
2. All of the 15 weather scenarios are equally probable in the next year.

Since the model was trained on weather data as well, we had a reasonably accurate prediction for each weather scenario. Based on the aforementioned assumptions, we calculated the next year’s predicted yield as the mean value of predicted yields for the 15 weather scenarios. We cannot know for sure what the weather is going to be like next year, but this approach allowed us to consider a number of possible outcomes. Another advantage of weather scenarios was that they allowed us to observe covariance between different varieties. Considering the significance of risk avoidance it is a good strategy to invest in decorrelated assets [17], i.e. to choose decorrelated varieties in the problem of seed selection. For each variety, there was a vector of predicted yields for each weather scenario. By comparing the yield vectors of two varieties we could infer if there is similarity or discrepancy in their performance in different weather scenarios. Covariance was calculated for each pair of varieties, effectively forming a covariance matrix.

### Portfolio optimisation

**Fig 3:**
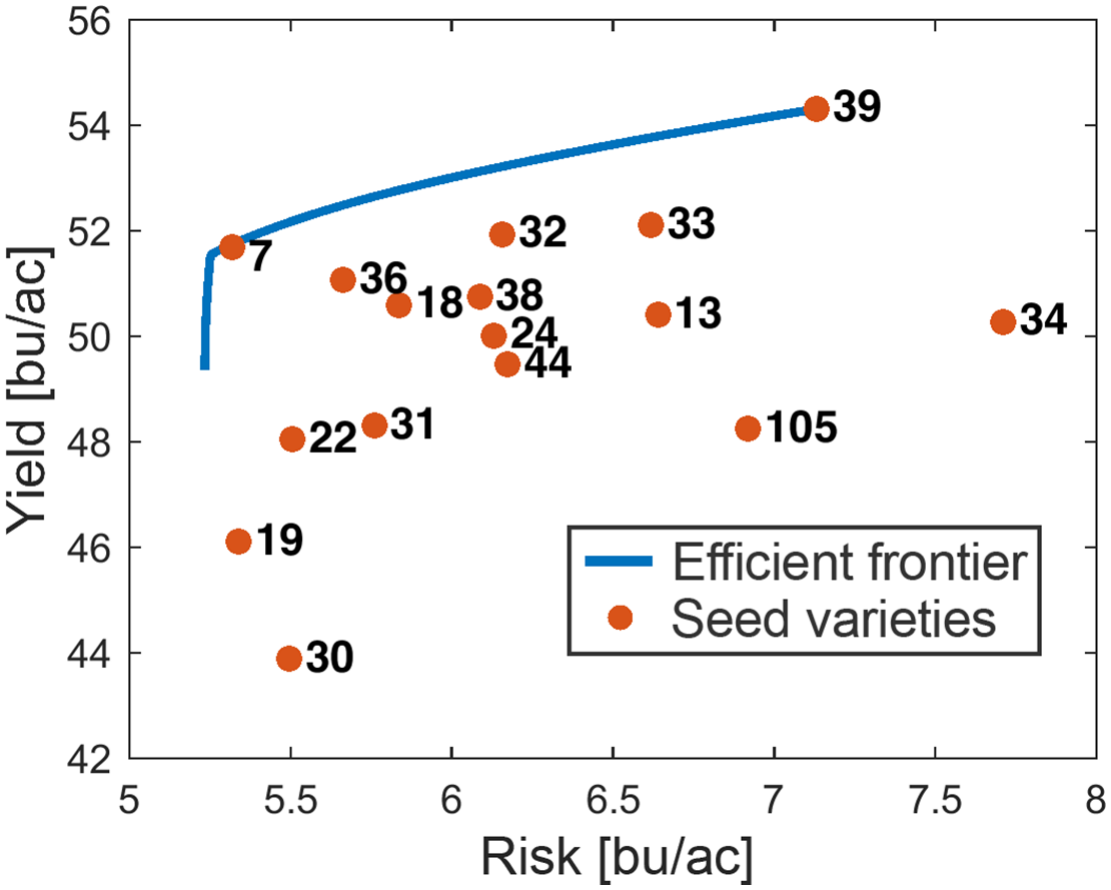
Example of efficient frontier. Blue line denotes the efficient frontier made of 100 Pareto-optimal portfolios. Red points stand for individual varieties.

The entries needed for portfolio optimisation are mean return of the assets and covariance matrix. In this case, we used predicted yield instead of the mean historical yield to better model the system and covariance matrix. They were calculated for each of 6,490 subregions. Next, portfolio optimisation was performed on the subregion level using these two inputs. Portfolio does not give a single optimal solution for the problem of selection, but a number of different solutions, located on the efficient frontier (Fig 3). Each point along this curve represents one Pareto-optimal portfolio. What this means is that for a given risk, there is no portfolio with a higher yield and, for given yield, there is no portfolio with a lower risk. The risk shown on the graphs is essentially the standard deviation of yield.

**Fig 4:**
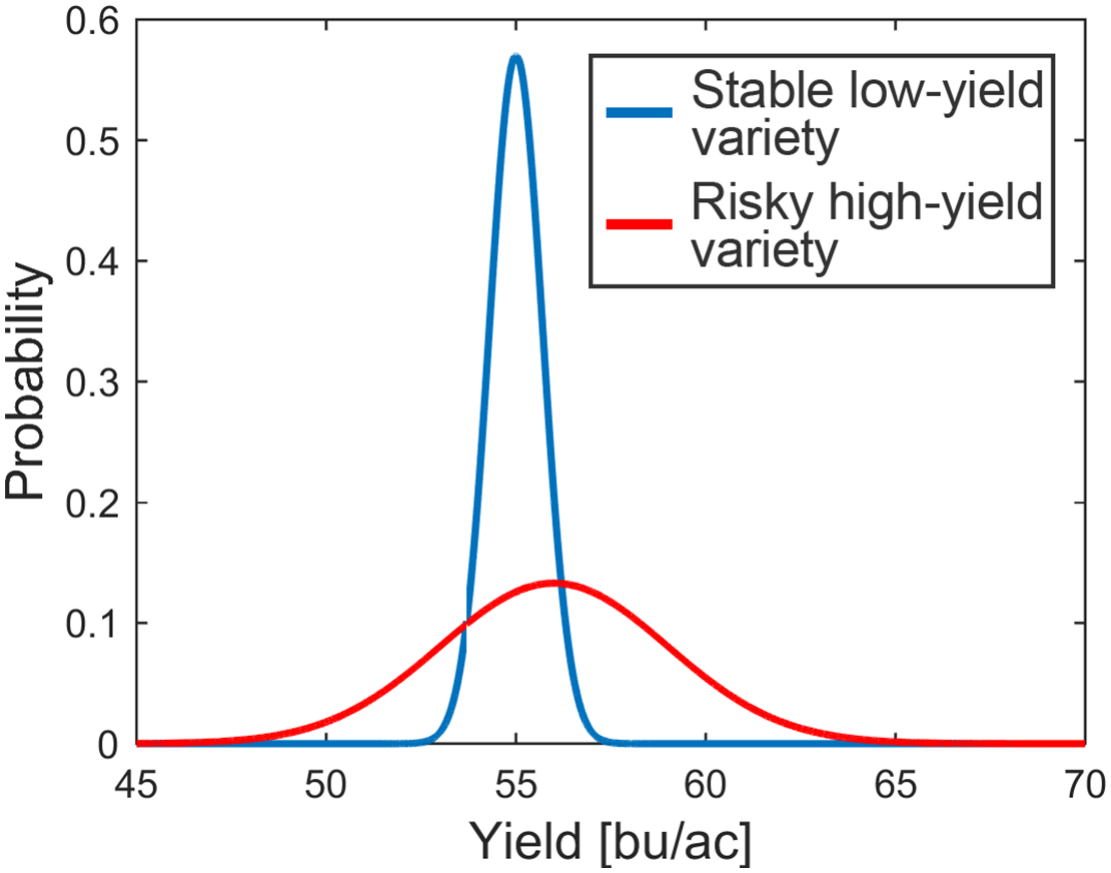
Two varieties with different predicted yield and risk. Blue curve shows a lower-yielding stable one and red shows higher-yielding risky one.

Difference between risky and high-yielding portfolios on one hand (red), and stable but lower-yielding ones on the other (blue) is illustrated in Fig 4.

**Fig 5:**
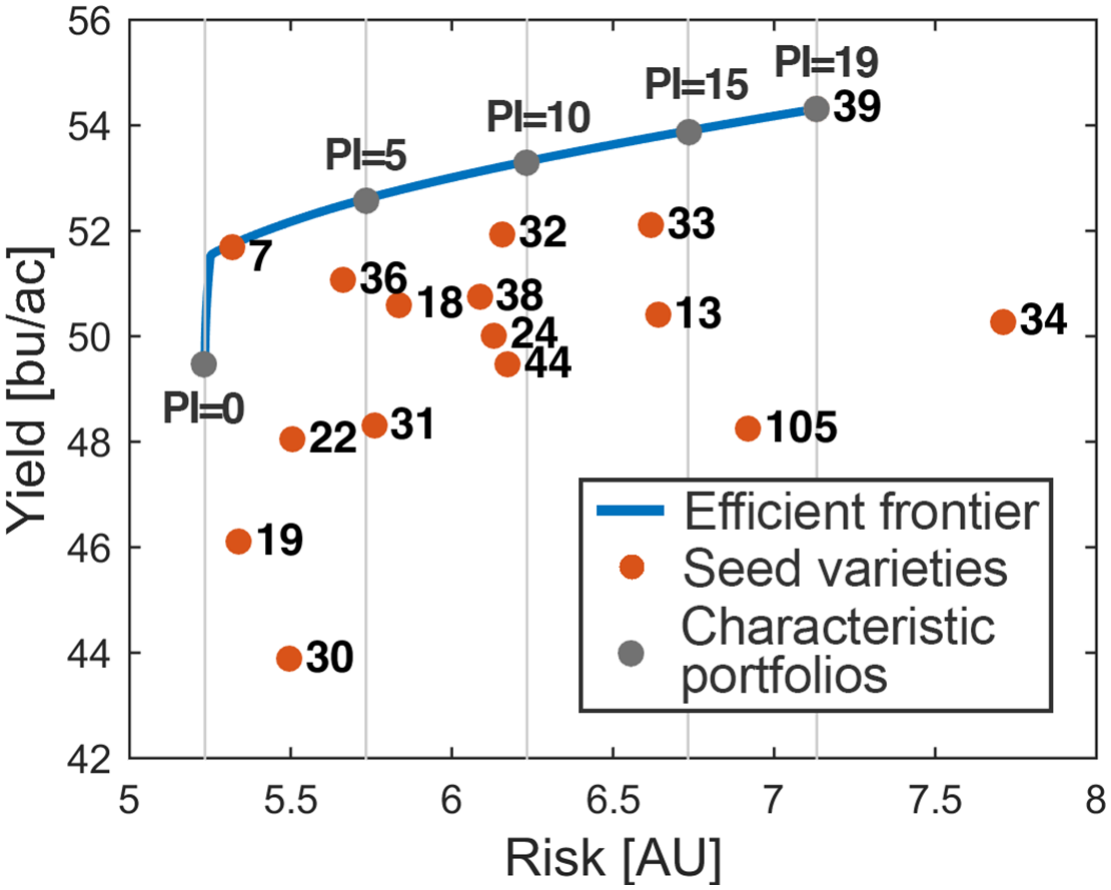
Efficient frontier divided into levels according to PI. Grey points signify portfolios for characteristic PI values.

This approach was particularly suitable for the problem stated in Crop Challenge because it leaves the possibility of choosing a different suboptimal portfolio for each characteristic profile of farmers in the region. Risk-averse farmers can therefore pick the low-risk portfolios, while more ambitious farmers can pick the risky, but high-yielding ones. PI value at a farm was a good measure of farmer’s willingness to take a risk. Farmers who have productive farms are less likely to fail than those who have less productive farms. Therefore, they can afford to take chances more than those with less productive farms, whose yield can be very disappointing if they take a risk. PI was thus incorporated in the portfolio optimisation as a measure of farmer’s risk aversion. Since the maximal value of PI was 19 and minimal 0, we divided the risk linearly into 20 levels between its minimal and maximal value on farms (Fig 5).

In this way, we obtained an optimal portfolio for every subregion. In the next step, we formed a 2D matrix, which contained percentual contribution *c*_*j*,*i*_ of each variety *i* in each subregion *j*, as in Table 3.

**Table 3:** Contribution of *N* varieties in portfolio of each subregion.

Location	Variety 1	Variety 2	…	Variety *N*
Subregion 1	*c*_1,1_	*c*_1,2_		*c*_1,*N*_
Subregion 2	*c*_2,1_	*c*_2,2_		*c*_2,*N*_
…			⋱	
Subregion 6490	*c*_6490,1_	*c*_6490,2_		*c*_6490,*N*_
$\sum_{1}^{6490}$	*x*_1_	*x*_2_	…	*x*_*N*_

Each row represents the optimal portfolio for the corresponding subregion. Contribution of a certain variety in the portfolio is denoted by *c*_*i*,*j*_, where *i* stands for subregion and *j* for variety. All contributions within a row must sum to 100%. Contributions of each variety were aggregated across the subregions, according to the *area* parameter, thus taking into account the size of agricultural land under soybeans inside a subregion. Lastly, the aggregated contributions were normalised, so that *x*_*i*_ stands for the percentage of variety *i* in the optimal portfolio for the whole Midwest region.

For every variety *i* the weighted average of contributions was calculated across the subregions (*x*_*i*_ in Table 3), with respect to the “area” parameter and then normalised, to keep the sum of *x*’s at 100% (Eq (1)). This parameter indicated the size of area under soybean inside a subregion and, the larger the area, the bigger share did it have in the sum.

$$\begin{array}{c} x_{i}=\frac{\sum_{j=1}^{6490}area_{j}\times c_{j,i}}{\sum_{\forall m,n}area_{m}\times c_{m,n}} \end{array}$$ (1)

Now, we had contributions of each variety in next year’s planting strategy and, lastly, varieties with contributions below 10% were discarded and the ones that remained were once again scaled to sum up to 100%.

## Results

Two parts of the solution were evaluated separately—yield prediction and seed selection. In the end, the complete algorithm was carried out and the resulting seed varieties are shown in the last section.

### Yield prediction

Different regression methods were validated using leave-one-year-out method and the results are shown in Table 4 in descending order, according to RMSE. Random forest proved to be the best in terms of both error and correlation coefficient. Therefore, we used it in all instances where yield prediction was needed.

**Table 4:** Comparison of different machine learning methods.

Classifier	RMSE [bu/ac]	MAE [bu/ac]	*r*	*ρ*
ANN	32.10	28.13	10.86%	12.83%
k-NN	14.10	11.09	18.15%	17.60%
MLR	11.25	8.97	18.75%	16.97%
SVM	11.04	8.73	15.96%	11.89%
WHR	10.49	8.23	33.16%	34.69%
**RF**	**10.43**	**8.21**	**37.51**%	**36.45**%

### Global sensitivity and uncertainty analyses

**Fig 6:**
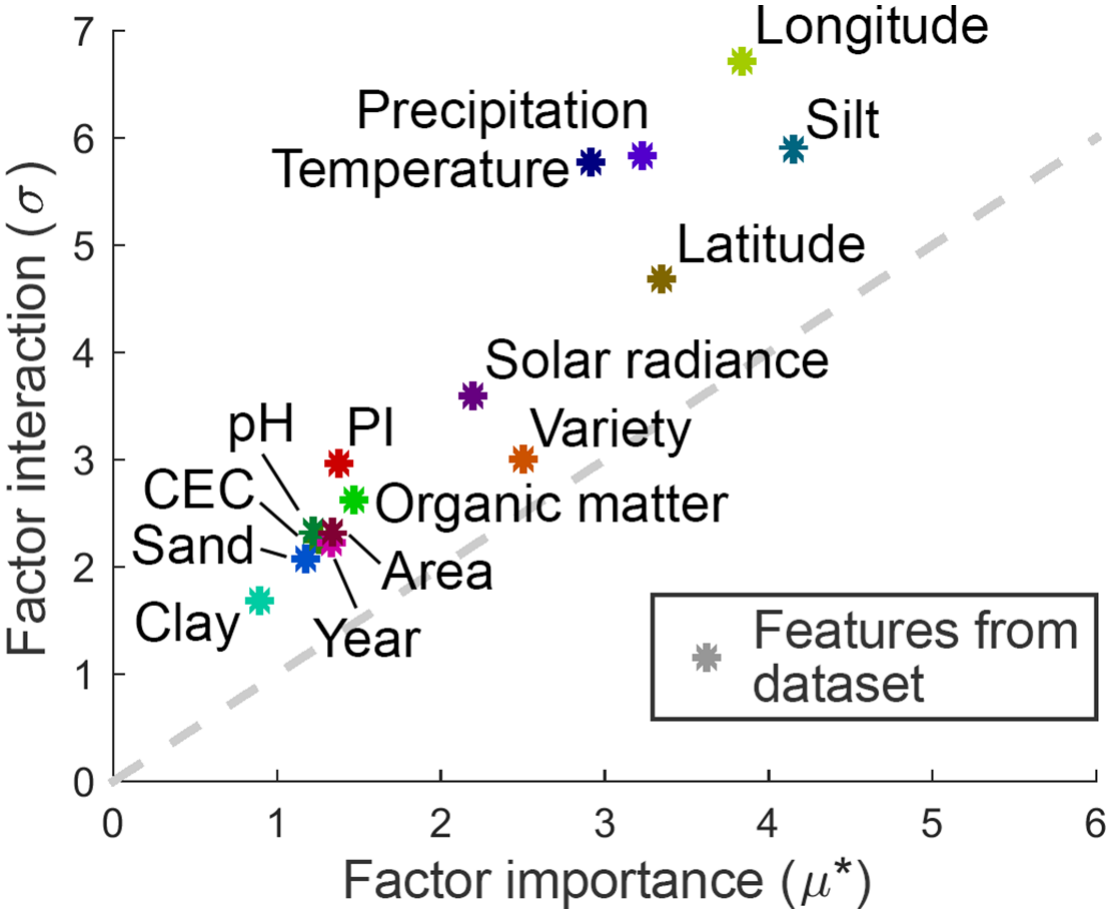
Results of Morris method. Horizontal axis represents the factor importance, i.e. the amount of direct influence of a factor on output, while the vertical axis represents factor interaction, i.e. the amount of influence in combination with other factors. Dashed grey line *σ* = μ* represents points with equal direct and indirect influence on output.

Global sensitivity and uncertainty analysis were performed using SALib, an open-source Python package [51]. The results of 160 Morris simulations (*k* = 15, *r* = 10) are shown in Fig 6.

All the factors are located above the dashed line, meaning that they influence the output through interaction with other factors more than on their own. Three clusters are visible in the graph. The first one contains factors with high importance (silt, longitude, latitude, precipitation, temperature), medium-importance factors (variety and solar radiance) are located in the second one, while the third one contains low-importance factors (organic matter, PI, area, year, pH, CEC, sand and clay). The first two clusters containing the 7 most important factors were selected for a more in-depth (Sobol’) analysis.

**Fig 7:**
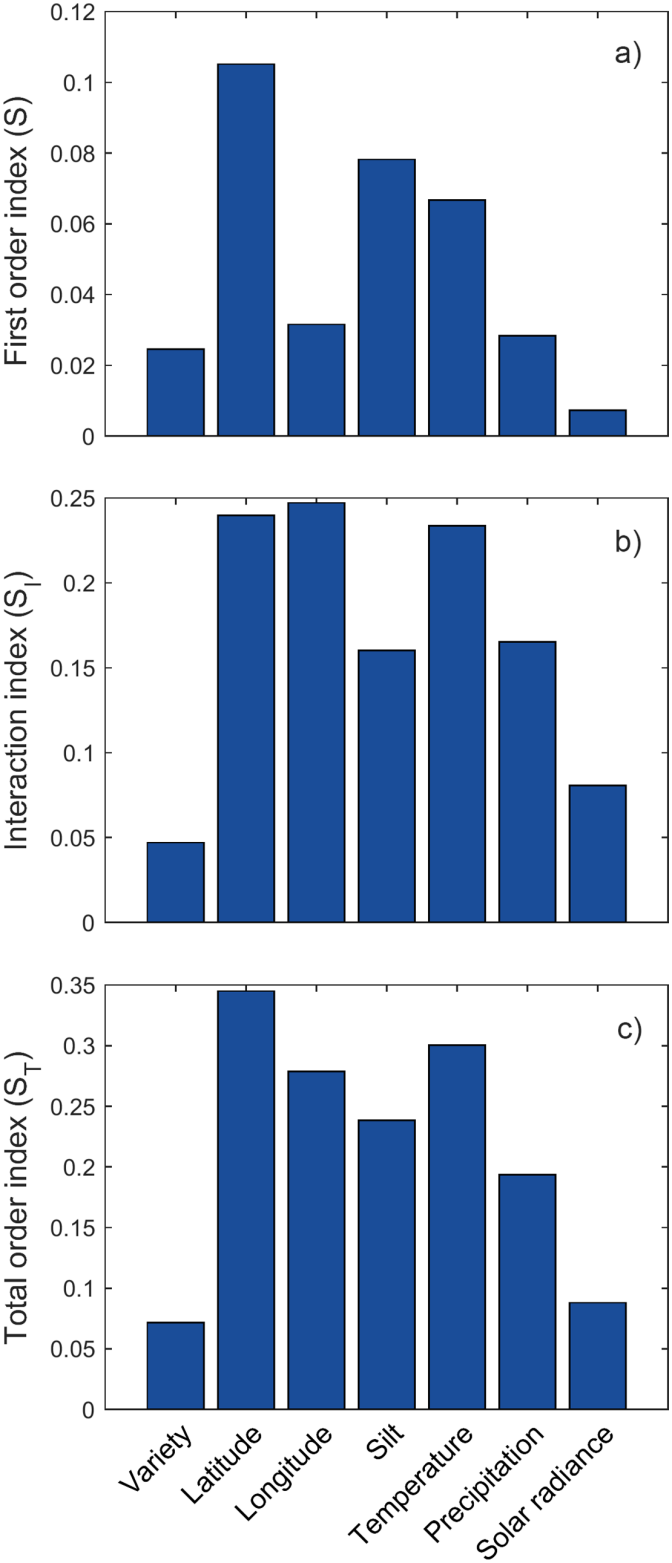
Results of Sobol’ method. a) First order index (direct influence on output); b) Interaction index (influence in combination with other factors); and c) Total order index (total influence on output).

Global sensitivity analysis was performed on the selected factors using 8192 Sobol’ simulations (*k* = 7, *M* = 512). The results are shown in Fig 7.

**Fig 8:**
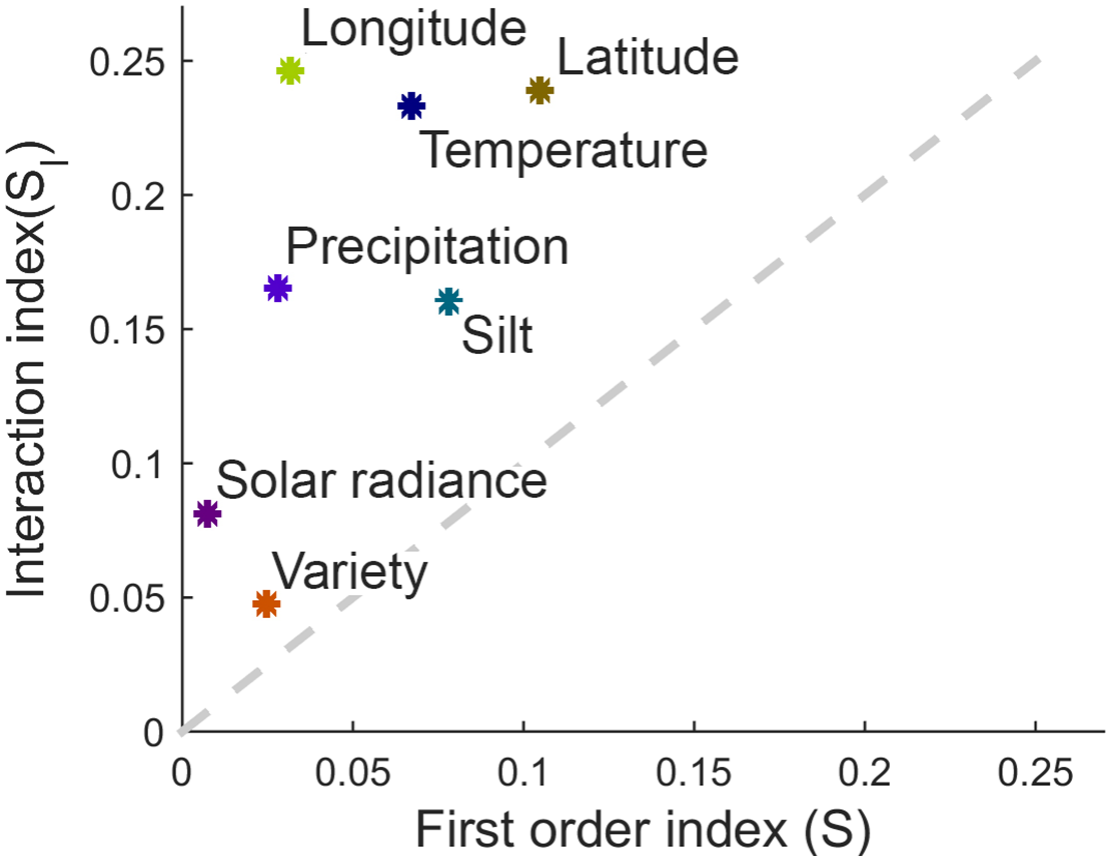
Indirect versus direct influence of factors on output. Indirect versus direct influence of factors on output, calculated using Sobol’ method. Horizontal axis represents the first order index, i.e. the amount of direct influence of a factor on output, while the vertical axis represents interaction index, i.e. the amount of influence in combination with other factors. Dashed grey line *S*_*I*_ = *S* represents points with equal direct and indirect influence on output.

As was the case with Morris method, the interaction between factors dominates over their direct influence on output (Fig 8). The ranking order of factors in terms of importance has slightly changed, but the overall sensitivity to factors is very similar.

**Fig 9:**
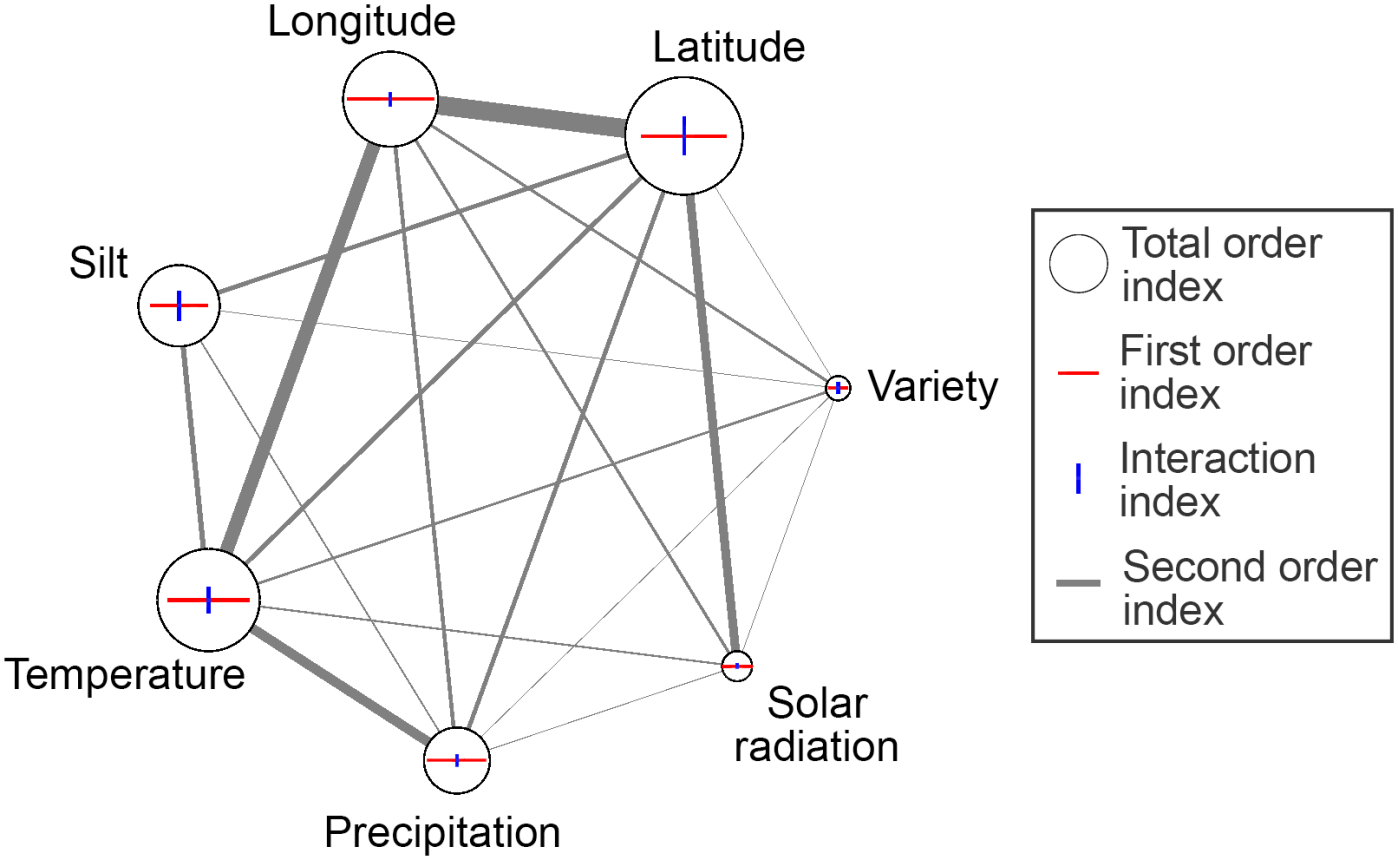
Sobol’ factor interaction. Circle diameter, red horizontal line length and blue vertical line length are proportional to total order, first order and interaction index, respectively. Second order index between two factors is proportional to the width of the grey edge that connects them.

One more advantage of Sobol’ over Morris method is that it allows for calculating the second order indices, which correspond to pairwise interaction between factors. A clear and intuitive visualisation of interactions, as proposed in [47], is shown in Fig 9.

**Fig 10:**
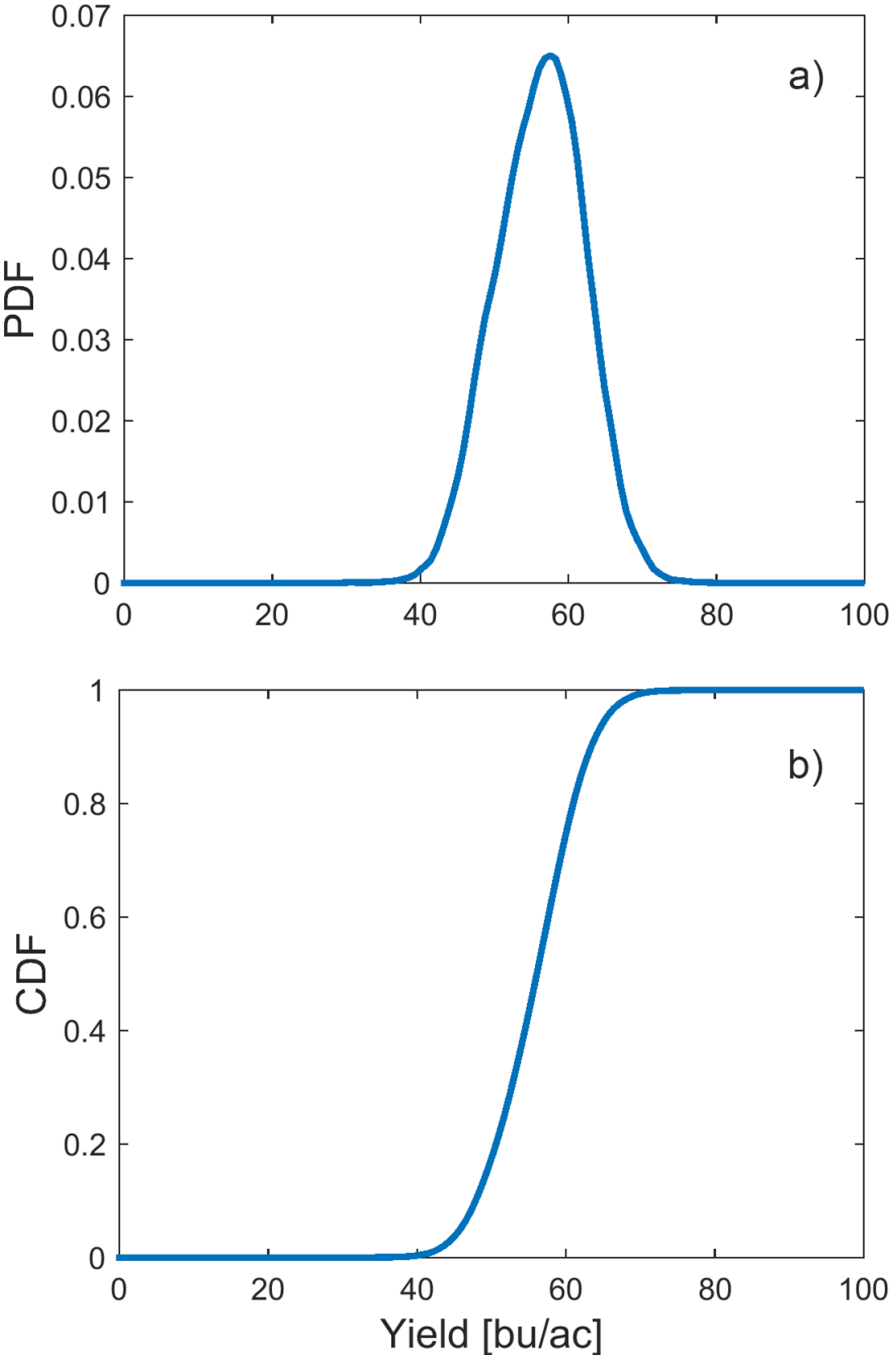
Results of uncertainty analysis. a) Probability density function (PDF); b) Cumulative distribution function (CDF).

For uncertainty analysis, we used the results of Monte Carlo simulations performed for Sobol’ global sensitivity analysis. Probability density function and cumulative distribution function (CDF) of the outputs are shown in Fig 10.

### Seed selection

Three different seed selection strategies were compared and their performances were measured on Experiment dataset:

1. Selection of the best variety
2. Selection using portfolio optimisation with farm’s PI value
3. Selection using portfolio optimisation with globally optimal risk threshold

Just as the yield prediction, they all were validated using leave-one-year-out method.

#### Selection of the best variety

Here, the strategy was to pick a single best variety for all subregions, or all experiment farms in the process of method evaluation. This is the most basic strategy, as it relies only on predicted yield and does not include portfolio optimisation. First, regression model was trained on six years and yield was predicted for the remaining year, but only for those farm-variety combinations for which the real yield was actually measured. In this way we could assess our selection by comparing the true yields of selected and non-selected varieties. Two vectors were formed—**p** and **r**. They both had as many elements as there were varieties planted in that year. Each element of vector **p** represented the predicted yield of a particular seed variety *i*. The predicted yield was actually the average value of predicted yields $\hat{y}$ across all farms where variety *i* was planted ($F^{i}$), weighted by the *area* parameter of these farms (Eq (2)).

$$\begin{array}{c} p_{i}=\frac{\sum_{j\in F^{i}}area_{j}\times {\hat{y}}_{j}^{i}}{\sum_{j\in F^{i}}area_{j}} \end{array}$$ (2)

Similarly to **p**, each element of vector **r** was the real yield *y* of seed variety *i*, again averaged across farms where this variety was planted ($F^{i}$) and weighted by the *area* parameter of farms (Eq (3)).

$$\begin{array}{c} r_{i}=\frac{\sum_{j\in F^{i}}area_{j}\times y_{j}^{i}}{\sum_{j\in F^{i}}area_{j}} \end{array}$$ (3)

The best variety was picked as the one that had the largest predicted yield, i.e. $v_{best}=\underset{i}{argmax}\left(p\right)$. As our goal was to pick the seed variety that was better than the others, real yield of this variety was compared to the mean real yield of all varieties planted in that year. Improvement *Q*, that selection of the best variety brought was, hence, calculated as the mean improvement that the best variety brought in all years.

$$\begin{array}{c} Q=\sum_{l=2009}^{2015}\frac{r_{l}\left[argmax\left(p_{l}\right)\right]}{mean(r_{l})} \end{array}$$ (4)

**Fig 11:**
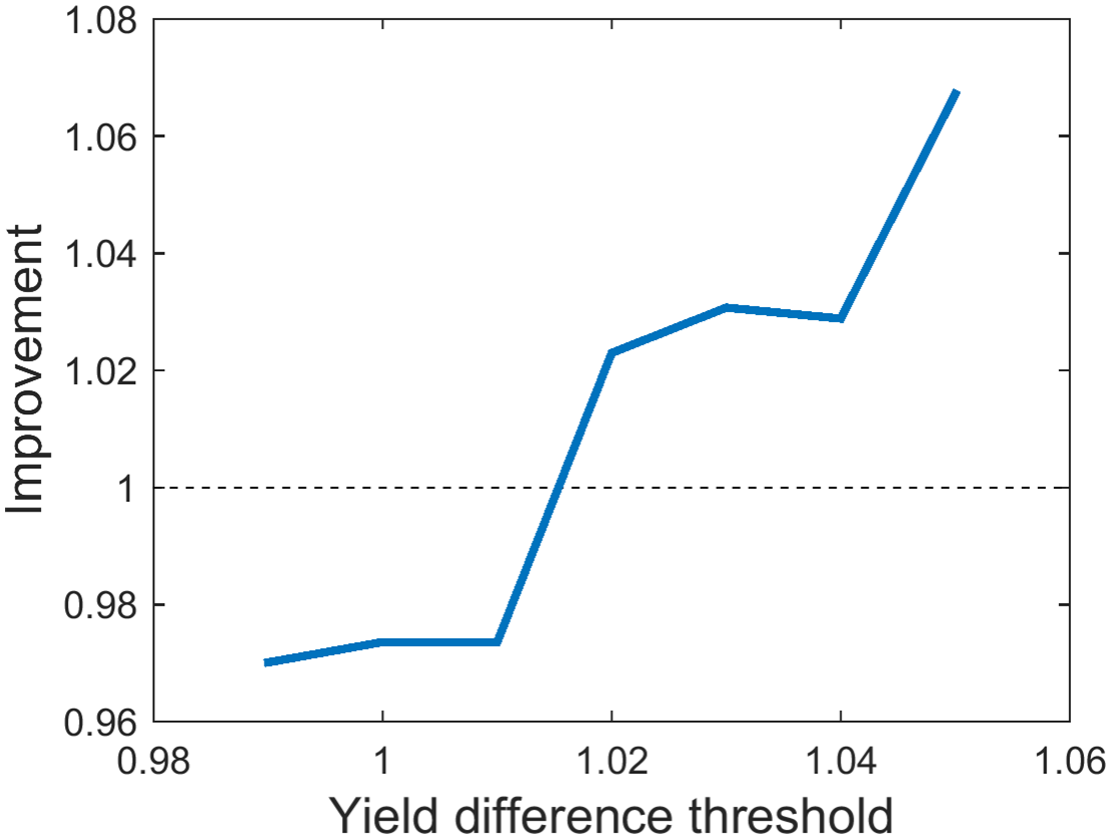
Improvement *Q* of the best variety method for different values of YDT.

The biggest improvement *Q* of +6.72% with the risk (standard deviation) of 7.52 bu/ac was achieved for YDT of 1.05 (Fig 11). Results for values of YDT between 0.8 and 0.99 are not shown because they are exactly the same as for 0.99.

#### Selection using portfolio optimisation with local risk threshold

With this strategy we assume that each farmer intends to plan the optimal selection of varieties for his/her farm, with respect to farm’s PI value. This is the realistic approach in which farmers choose optimal solutions for their own farms without having concerns about the global profit of farmers in the region.

**Fig 12:**
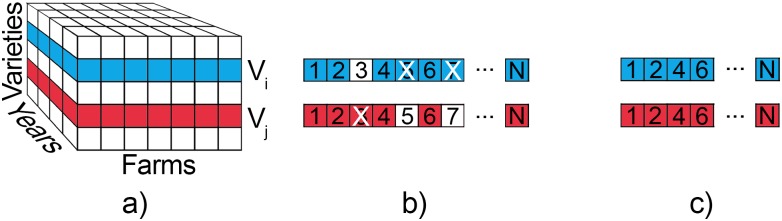
Calculating covariance matrix from the “prediction cube”. a) Prediction cube; b) 2D matrices vectorised; c) Remaining data after removal of incomplete pairs.

We aggregated the contributions of varieties across all farms and picked the ones that bring the best overall improvement. Besides the predicted yield, portfolio optimisation requires covariance matrix as an input. There were two problems with the covariance matrix. The first was that it could not be calculated using just the historical data. Some pairs of varieties had never been planted on the same farm or even in the same year. For this reason, covariance was calculated on predicted yields. This meant that for each variety in the test year, we needed to have its samples in the training dataset, so that we could train the regression model for that particular variety. There were varieties that were only planted in one year. For these reasons, we calculated the covariance matrix on predicted yields for all years, without validation, in the following way. We predicted the yield for all possible combinations of years, farms and varieties, for which this was feasible, thus forming a “prediction cube” (Fig 12a). Covariance of a pair of varieties was calculated as the covariance between two corresponding layers of the cube. These layers were essentially matrices that consisted of predicted yields of one variety in all farms and all years. The matrices were vectorised (Fig 12b) and if one of them had an empty value (white square in Fig 12b), this element was removed from both vectors (crossed squares in Fig 12b). Finally, covariance between two varieties was calculated as covariance between the two resulting vectors (Fig 12c).

A table similar to Table 3 was formed. The only difference was that instead of 6490 rows for each subregion, there was a row for each of the *M* farms in test year (Table 5).

**Table 5:** Contribution of *N* varieties in portfolio of each farm.

Location	Variety 1	Variety 2	…	Variety *N*
Farm 1	*c*_1,1_	*c*_1,2_		*c*_1,*N*_
Farm 2	*c*_2,1_	*c*_2,2_		*c*_2,*N*_
…			⋱	
Farm *M*	*c*_*M*,1_	*c*_*M*,2_		*c*_*M*,*N*_
∑	*x*_1_	*x*_2_	…	*x*_*N*_

Contributions were aggregated across farms.

Up to five best varieties were selected with contributions of more than 10% and the others were discarded. Contributions of the varieties that left (${\hat{v}}_{1},{\hat{v}}_{2},...,{\hat{v}}_{K},1\leq K\leq 5$) were once again normalised to add up to 100%. Portfolio yield in year *l* (*Y*_*l*_) was then calculated as

$$\begin{array}{c} Y_{l}=\sum_{k=1}^{K}y_{k}\times {\hat{x}}_{k}, \end{array}$$

where *y*_*k*_ is the real yield of each variety ${\hat{v}}_{k}$ in year *l* and ${\hat{x}}_{k}$ is variety’s contribution in portfolio after normalisation.

Just as with the strategy of picking one most promising variety, improvement *Q* that this approach brought was calculated as the mean improvement over the years that portfolio optimisation brought comparing to the mean yield *m*_*l*_ in the corresponding year (Eq (5)).

$$\begin{array}{c} Q=\sum_{l=2009}^{2015}\frac{Y_{l}}{m_{l}} \end{array}$$ (5)

**Fig 13:**
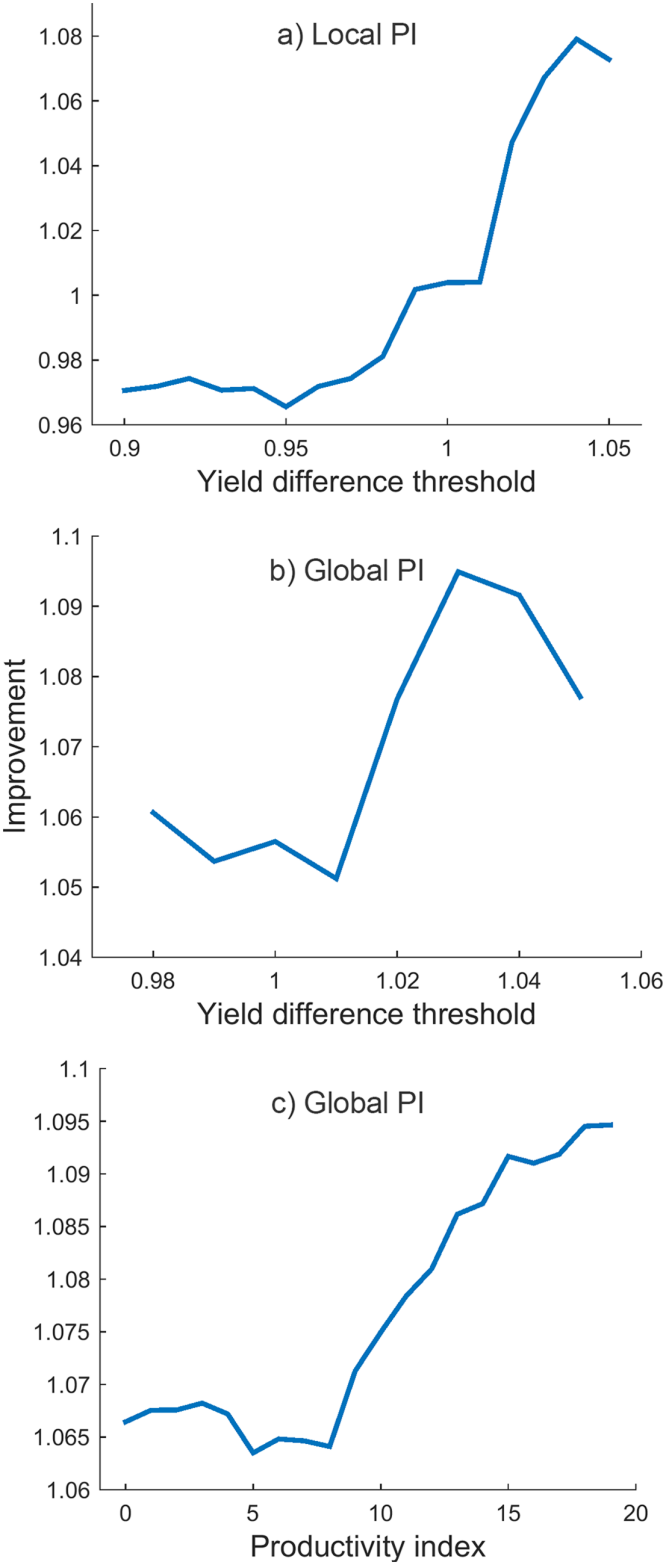
Improvement *Q* of portfolio optimisation approaches with different values of YDT and PI. a) Local PI (YDT is varied, PI takes local values); b) Global PI (YDT varied, PI fixed); c) Global PI (YDT fixed, PI varied).

This approach was the one used on the region dataset for getting the optimal selection of varieties for the whole region, considering each subregion’s PI. In the process of strategy evaluation, subregions were substituted by farms and hence instead of subregions’ PIs we chose portfolios according to farms’ PIs. The optimal value of YDT was obtained by brute force optimisation. This parameter took values between 0.8 and 1.05 and 1.04 proved to be the optimal value. Fig 13a shows the objective function between 0.9 and 1.05 with step 0.01. For YDT below 0.9 the function had the same value as for 0.9 and for values greater than 1.05, *Q* could not be calculated, because too many varieties were discarded, leaving certain years without any varieties that the algorithm could select. The optimal value was 1.04 and it brought yield increase *Q* of 7.91%, with risk of 7.60 bu/ac.

#### Selection using portfolio optimisation with globally optimal risk threshold

The goal of this approach, with globally optimal PI, was to show the full potential of portfolio optimisation in terms of yield increase. In this case we show what the yield would be like if seed selection was centrally managed for the whole region. Besides YDT, another parameter that was varied was PI, which was now the same for all farms. Optimal YDT and PI values were found using brute force search, as the ones which gave the highest *Q*. As with the previous method, YDT was chosen from range 0.8 to 1.05 with step 0.01, while PI took the values from 0 to 19, with step 0.25. The optimal YDT proved to be 1.03, while the optimal PI was 18.25. With these parameters, yield improvement was 9.53% and risk 7.96 bu/ac. The objective functions where one parameter is fixed and other varied are shown in Fig 13b and 13c.

#### Final selection

In order to give the answer to the problem stated in the Syngenta Crop Challenge, we analysed the Region dataset and inferred the optimal selection of seeds for the American Midwest. According to the methodology elaborated in this work, we predicted the yield, performed portfolio optimisation and aggregated the contributions of varieties across all subregions. We performed portfolio optimisation using local PI values and optimal YDT for this method, as it is the best solution for a region with a multitude of farmers who select seed varieties individually, on a local scale. This left us with four varieties shown in Table 6 along with their percentages in the final selection.

**Table 6:** Final selection of seeds for the whole region.

Variety	Percentage
${\hat{v}}_{1}$	65%
${\hat{v}}_{2}$	12%
${\hat{v}}_{3}$	12%
${\hat{v}}_{4}$	11%

**Fig 14:**
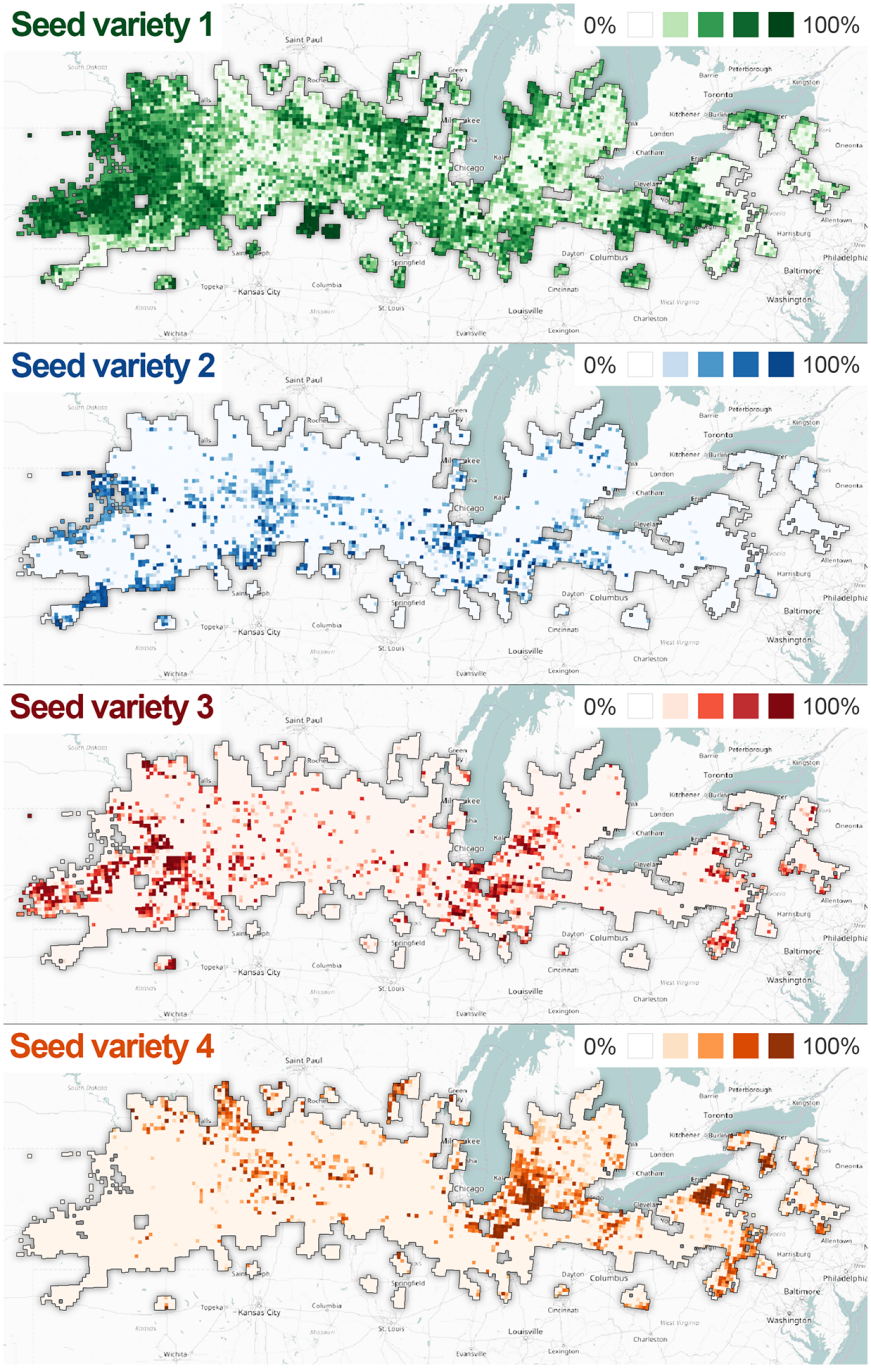
Regional distribution of selected soybean varieties. Distributions of ${\hat{v}}_{1}$—green, ${\hat{v}}_{2}$—blue, ${\hat{v}}_{3}$—red and ${\hat{v}}_{4}$—brown.

From the point of view of seed distributors it is very important to know what will be the demand for particular seed varieties not only across the whole region, but also on a smaller scale. In that way they could plan their supplies for different distribution centres. Based on the results of seed selection they can have an insight in which varieties will be needed in certain area and in what amount. They can adjust their supplies accordingly and thus minimise transport of seeds between the distribution centres and avoid situations where they are in lack of some seed variety or have a huge stockpile of soybean seeds that they cannot sell. The spatial distribution of the selected seed varieties was drawn in QGIS [52] over the OpenStreetMap layer [53] (Fig 14).

### Discussion and conclusion

The aim of this work was to develop a strategy for selection of soybean seed varieties. The selected varieties would be distributed across the Midwestern United States before the planting season. Further transport of seeds between storage and distribution centres should be as low as possible.

First, we needed to predict the yield of different varieties across the region. For the purposes of training and testing of regression algorithms we used the Experiment dataset, which, besides weather and soil data, included the yield. Due to its robustness to diverse data and efficiency in handling categorical data, such as seed variety, random forest proved to be the best algorithm in terms of both error and correlation coefficient. Some had issues with non-linearity of data (MLR and SVM), while others could not handle well the high dimensionality of the problem (k-NN and SVM).

Modern machine learning gives far better results than traditional regression techniques, such as linear or higher-order polynomial regression. However, the problem with them is that, due to high non-linearities, it is impossible to trace the interaction between input parameters and their individual influence on output. Global sensitivity and uncertainty analyses provide the means for exploring the “black box” model and explaining the mechanism behind it. Uncertainty analysis showed that the outputs of the prediction model are distributed following a unimodal distribution with a great deal of resemblance to Gaussian function, which is in accordance to the empirically acquired yield distributions. Results of Morris method reveal that soil parameters are generally less important than others, with exemption of silt content, which is one of the most important factors, along with the location of the farm and weather parameters. Sobol’ analysis gives a more precise insight in the sensitivity of the model to the 7 factors chosen after screening. Latitude proved to be the most important feature, which is in line with classification of soybean seeds according to their relative maturity group. Namely, the US territory is divided into belts stretching from east to west, where the recommended relative maturity group changes with the latitude of the belt. Later-maturing varieties require longer time to advance through growth stages. This gives them advantage over the sooner-maturing varieties, as they are left with more time to utilise solar energy and exploit minerals from the soil, thus usually achieving higher yields. However, their downside is that the longer growing season imposes the risk of being hit by an early frost or even snow, which is why they are usually planted in southern parts of the US. It would be very beneficial to include relative maturity group of varieties in the analysis, from points of view of both yield prediction and risk assessment. As this kind of information was not included in the dataset, we were able to include this aspect of growing soybean only indirectly, through relationship between variety and latitude. The second most important feature is temperature. Soybean grows best in warm weather, but warm weather also requires enough of water for evapotranspiration of plants. We assume that this principle is encapsulated in substantial interaction between temperature and precipitation. Even though longitude on itself may not be the most important factor, the strongest interactions are, in fact, witnessed between it on one side and temperature and latitude on the other. This is probably due to the fact that climate is directly related to the position of the farm, i.e. the further it is to the west and north, the lower the temperatures are. Another relationship, between farm’s location and weather, is observed through the considerable interaction of latitude and solar radiance, which is in accordance with the fact that in temperate climate zones southern areas generally receive more sunlight than areas in the north. As for the soil parameters, except the silt content, none of them proved to be very indicative of yield. This could be a consequence of the soybean properties, i.e. that soybean grows well in all soils, but could also be a consequence of bias in the dataset. Farms may have been chosen specifically for their soil parameters, which are optimal for growing soybean, so there may be a lack of soil variability in the dataset. High importance of temperature, precipitation and solar radiance witnessed in the analysis means the yield was highly affected by weather conditions, thus justifying the weather scenarios approach. Lastly, although the choice of variety was not as important for the model as were the other parameters, it is interesting to note that it had interaction with every other feature. This means that the relationship between genotype and the environment is very complex and that there are various factors influencing its growth. Genotype-environment interaction in agriculture is usually analysed by applying AMMI (additive main effects and multiplicative interaction) model [54, 55] on data from randomised block experiments. This model, however, was not suitable for the dataset that we had at disposal, mainly due to the sheer size of the data. There were 174 genotypes planted on some of 205 farms in 7 years, which is an exceptionally large number of both genotypes and environments to be considered. Furthermore, the dataset was unbalanced, i.e. not all varieties were planted in all years on all farms. Thus, due to the missing genotype-environment combinations, modified version of the algorithm would have to be used. Another problem with AMMI analysis is that it is oriented towards concluding which environment a genotype is suitable for, while in this research we needed to precisely predict the yield of each variety in each environment, so that in the next step portfolio optimisation could be performed.

It would also be interesting to include genetic information about varieties in this study. It could reveal which parts of the DNA are responsible for particular plant traits, pest resistance or drought tolerance. Furthermore, this would lead researchers towards quicker and more efficient breeding of new varieties. As DNA sequences were not included in the dataset, the performance of varieties in different weather and soil conditions was inferred from the data using machine learning. Thus, it was machine learning models that represented the knowledge acquired about the relationship between genotype and the environment. Similar dataset which included genetic information was provided also by Syngenta, through another challenge, Syngenta AI Challenge [56]. However, the varieties analysed in the two challenges are not the same, so the datasets are not complementary and thus require a separate analysis.

Based on predicted yields, three different approaches to seed selection were tested. The first one was to select just the best variety, i.e. the one with the highest predicted yield. Other two approaches included portfolio optimisation of varieties for each farm using local PIs and one globally optimal PI, respectively. Contribution of each variety in portfolios was aggregated for all farms and the final portfolio of up to five best varieties was constructed. The yield of the final portfolio was then compared to the mean yield of seed varieties. The results for all three approaches are shown in Table 7.

**Table 7:** Comparison of methods for variety selection.

Method	*Q*	Risk [bu/ac]
The best variety	6.72%	7.52
Portfolio optimisation (local PI)	7.91%	7.60
Portfolio optimisation (global PI)	9.53%	7.96

The best variety approach served as a benchmark. It had a slightly lower risk than the other ones, but the improvement was lower as well. However, the biggest drawback of this approach is that it does not allow the farmer to define the exact amount of risk he or she can take. This was overcome by the portfolio optimisation approach, where the local PIs were used. The risk increased for negligible 1%, while the improvement increased for 17% comparing to the benchmark, thus justifying the use of portfolio optimisation in seed selection. At last, the global PI approach showed the full potential of portfolio optimisation in this problem. It brought the biggest improvement, 26% above the benchmark, with the increase in risk of just 6%. It showed that if all farms had their seeds chosen according to some global strategy, the whole region could prosper. Although this approach would have many practical obstacles, due to different risk profiles of farmers and possibly unfair centralised decision system, its potential should be further investigated, possibly with the involvement of insurance companies. Another subject of future work should be development of a more accurate yield prediction model. Although the one used in this work did manage to serve as a good basis for the seed selection algorithm, there is still room for improvement. It could be achieved either by using a larger dataset or by incorporating the agricultural knowledge in regression, possibly through the use of graphical models. Research could also be continued in the direction of choosing the optimal soybean varieties for the future considering the effects of climate change. Their performance will definitely be affected by the global temperature rise and changing weather patterns, so this can be a good starting point for long-term planning. One more aspect that could be covered in further studies is variety’s resistance to pests. Such information could be included in portfolio optimisation, as resistance to diseases is an important contributor to the risk associated to a variety. Furthermore, the risk could be spatially adjusted, according to the spread of a disease throughout the Midwest, so that the more resistant varieties are selected at locations where disease is more likely to strike. However, the available dataset did not include information about the resistance to diseases. Finally, future work could be concentrated around other decision variables besides yield. The additional variables could include nitrogen-fixation ability, protein and oil content of produce and its price on the market. As was the case with the resistance to diseases, this type of analysis requires additional data, which we did not have at our disposal.

Considering the capability of our method to select appropriate high-yielding seed varieties for the region based on the state-of-the-art analytics applied to soil and climate data, we believe that this work has a huge practical as well as scientific value and that it was an important contribution to Syngenta Crop Challenge.

## Supporting information

S1 Fig Histogram of yield. Histogram of yield across years, farms and varieties.
(TIF)

S2 Fig Histogram of yield / check-yield ratio. Histogram of yield / check-yield ratio across years, farms and varieties.
(TIF)

S3 Fig Histogram of years. Histogram of years in which samples were collected.
(TIF)

S4 Fig Histogram of farms. Histogram of farms at which samples were collected. Farms are represented with numbers from 1 to 205.
(TIF)

S5 Fig Histogram of varieties. Histogram of varieties across years and farms. Varieties are represented with numbers from 1 to 174.
(TIF)

S6 Fig Histogram of cation exchange capacity. (TIF)

S7 Fig Histogram of organic matter content. (TIF)

S8 Fig Histogram of pH. (TIF)

S9 Fig Histogram of clay content. (TIF)

S10 Fig Histogram of silt content. (TIF)

S11 Fig Histogram of sand content. (TIF)

S12 Fig Histogram of cumulative temperature. (TIF)

S13 Fig Histogram of cumulative precipitation. (TIF)

S14 Fig Histogram of cumulative solar radiation. (TIF)

S15 Fig Histogram of the “Area” parameter. (TIF)

S16 Fig Histogram of soil productivity index. (TIF)

S17 Fig Box plot of yield throughout the years. (TIF)

S18 Fig Average yield at farms. Yield was averaged across years and varieties.
(TIF)

S19 Fig Average yield per variety. Yield was averaged across years and farms.
(TIF)
