# Supplementary figures and images for: Portfolio optimization for seed selection in diverse weather scenarios
Source: PLoS One. 2017 Sep 1;12(9):e0184198. doi: 10.1371/journal.pone.0184198 (PMC5580993; doi:10.1371/journal.pone.0184198)

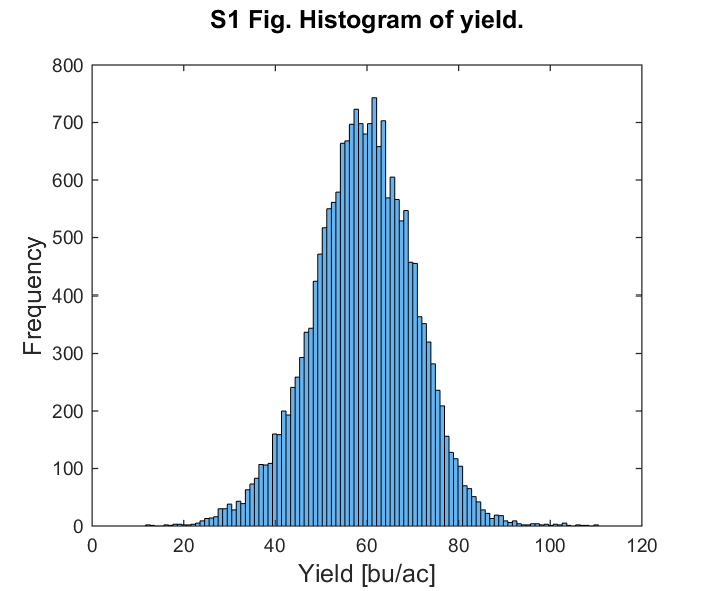

Supplement: S1 Fig — Histogram of yield across years, farms and varieties. (TIF) [file pone.0184198.s001.tif]

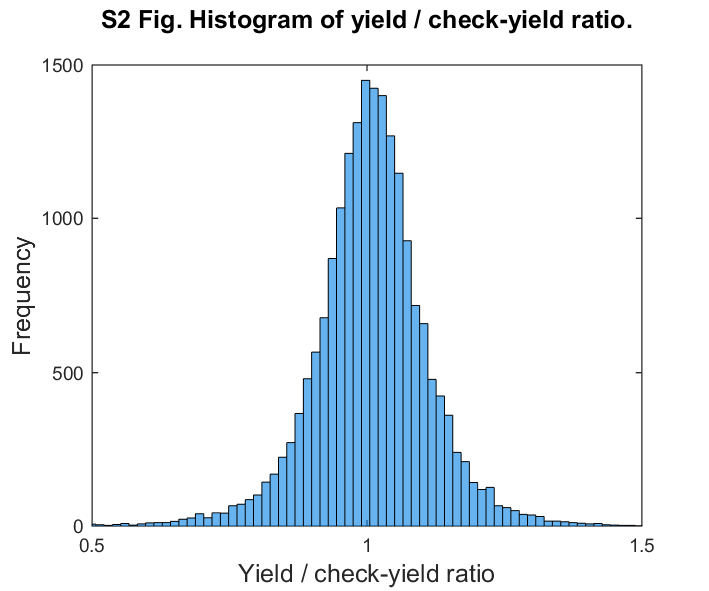

Supplement: S2 Fig — Histogram of yield / check-yield ratio across years, farms and varieties. (TIF) [file pone.0184198.s002.tif]

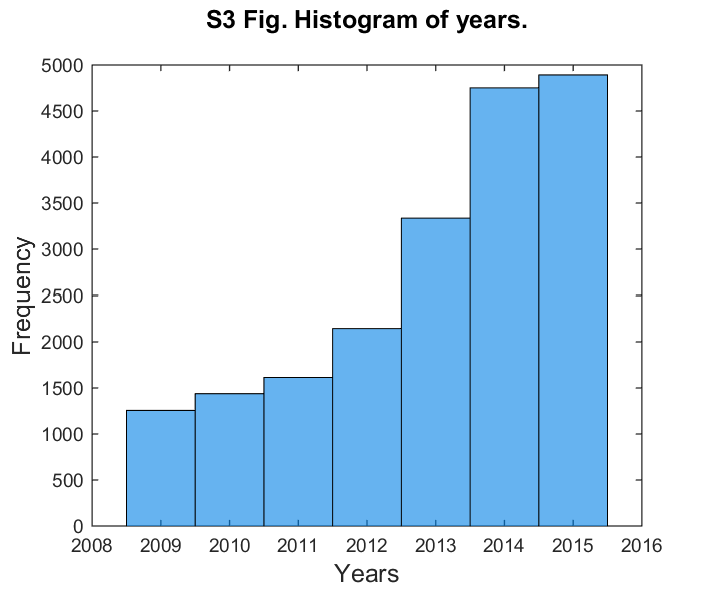

Supplement: S3 Fig — Histogram of years in which samples were collected. (TIF) [file pone.0184198.s003.tif]

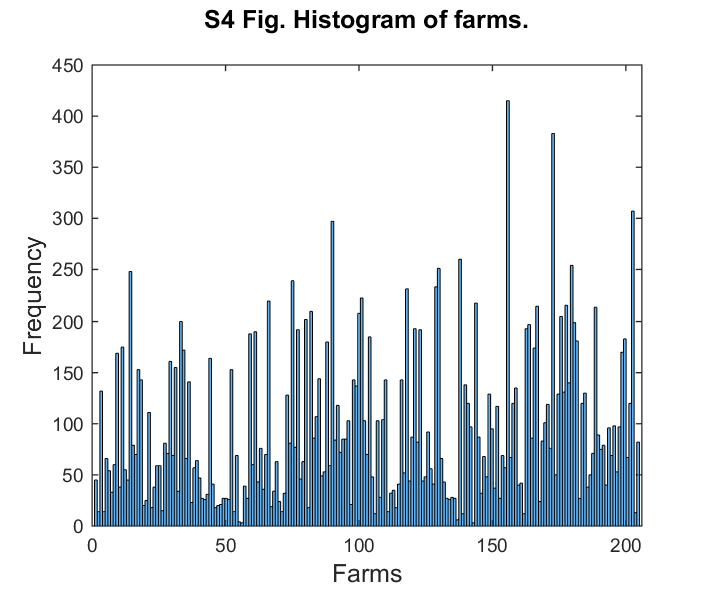

Supplement: S4 Fig — Histogram of farms at which samples were collected. Farms are represented with numbers from 1 to 205. (TIF) [file pone.0184198.s004.tif]

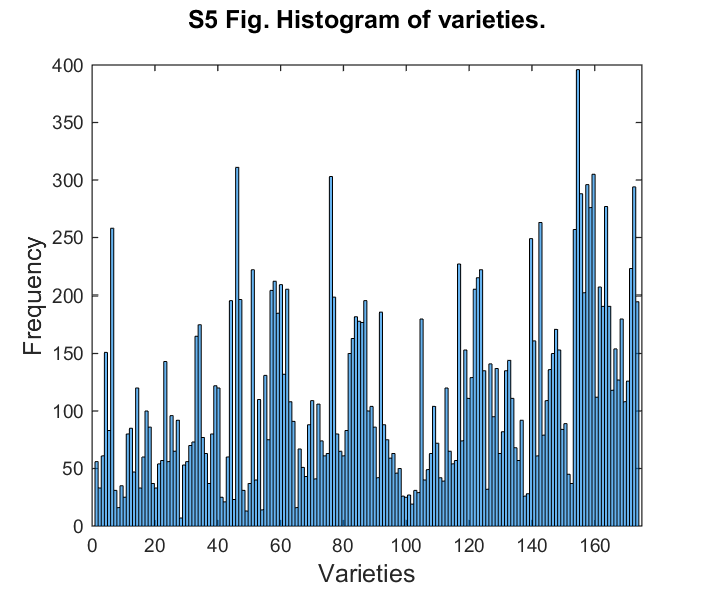

Supplement: S5 Fig — Histogram of varieties across years and farms. Varieties are represented with numbers from 1 to 174. (TIF) [file pone.0184198.s005.tif]

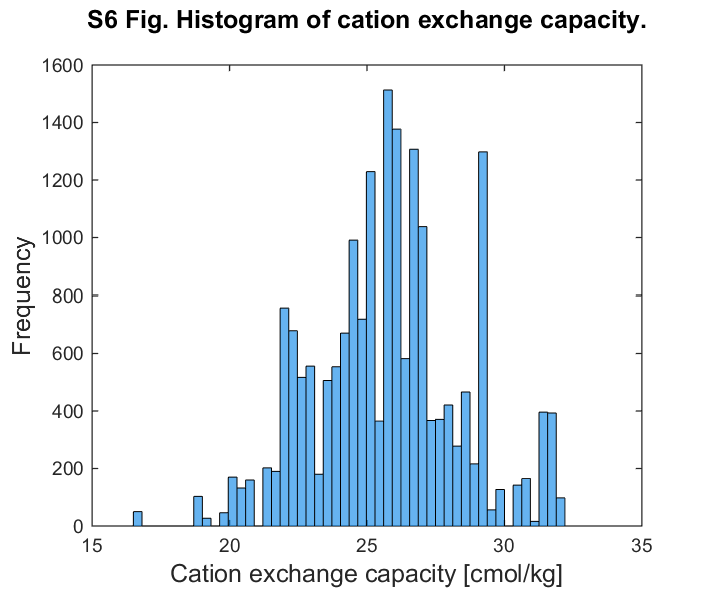

Supplement: S6 Fig — (TIF) [file pone.0184198.s006.tif]

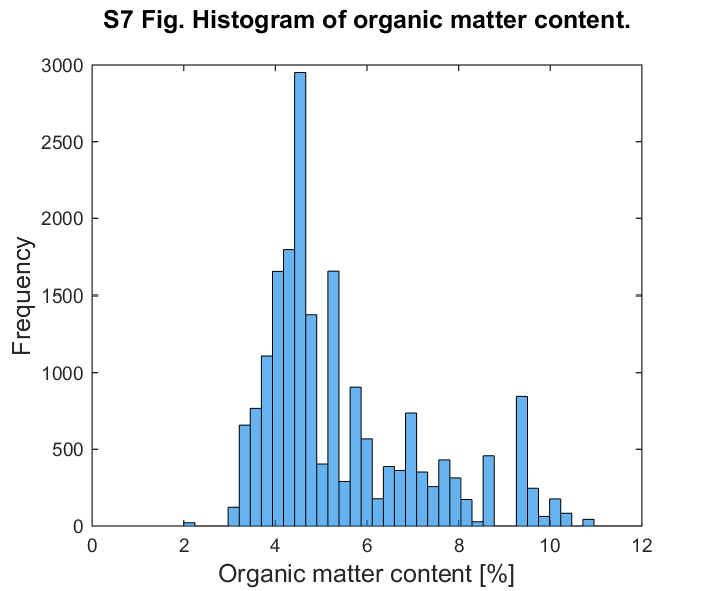

Supplement: S7 Fig — (TIF) [file pone.0184198.s007.tif]

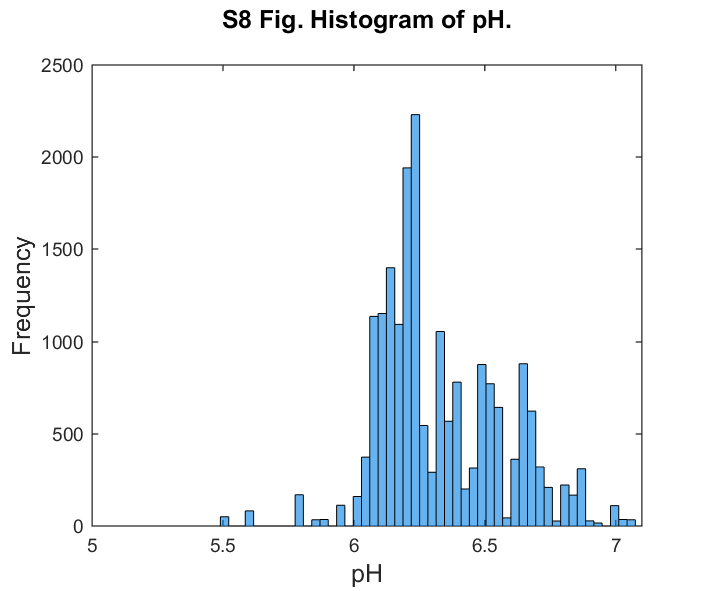

Supplement: S8 Fig — (TIF) [file pone.0184198.s008.tif]

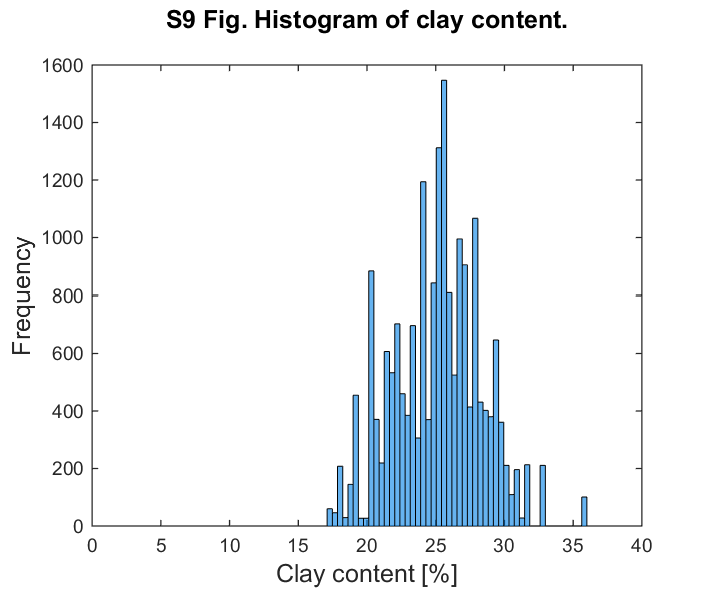

Supplement: S9 Fig — (TIF) [file pone.0184198.s009.tif]

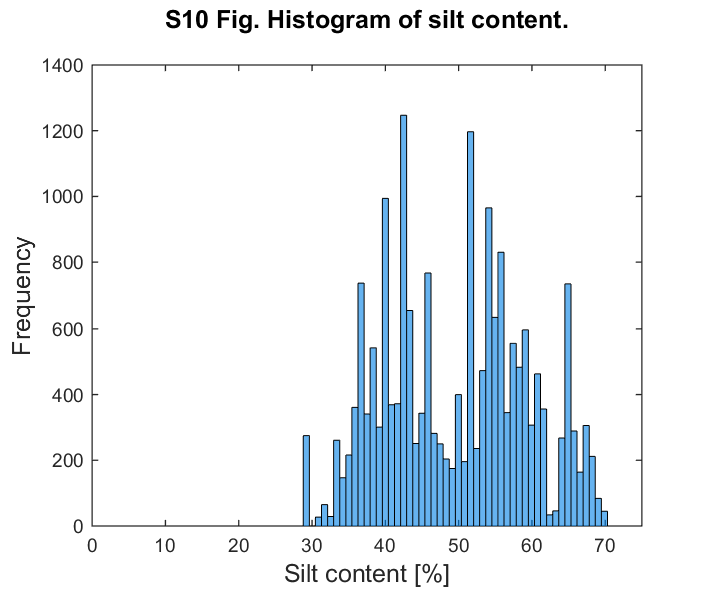

Supplement: S10 Fig — (TIF) [file pone.0184198.s010.tif]

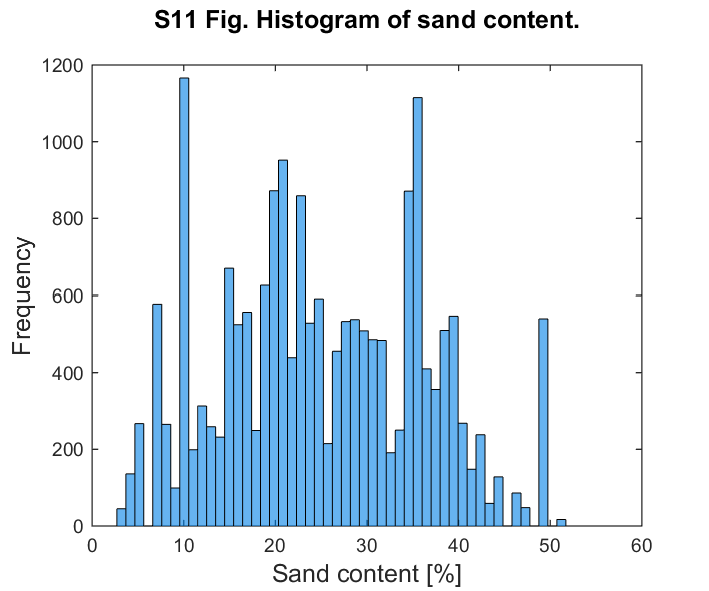

Supplement: S11 Fig — (TIF) [file pone.0184198.s011.tif]

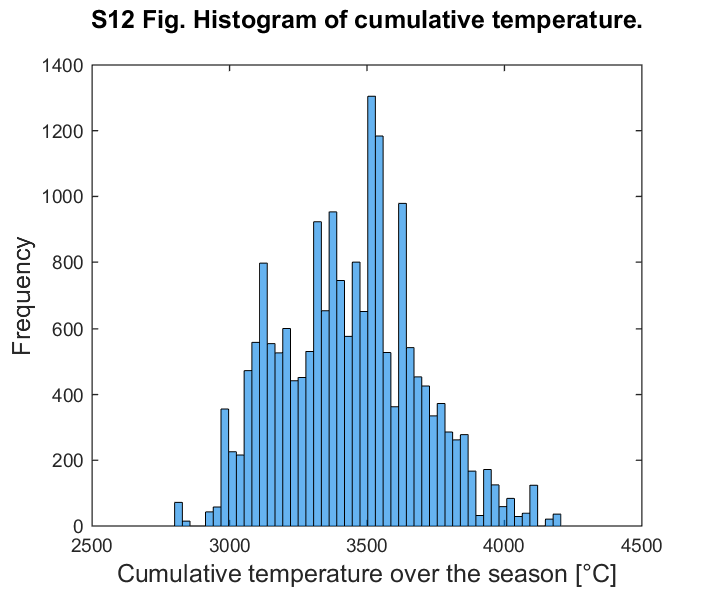

Supplement: S12 Fig — (TIF) [file pone.0184198.s012.tif]

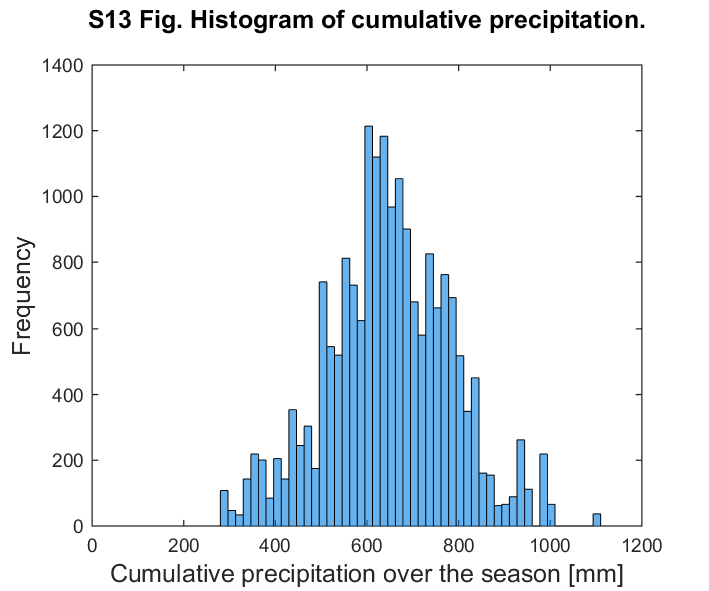

Supplement: S13 Fig — (TIF) [file pone.0184198.s013.tif]

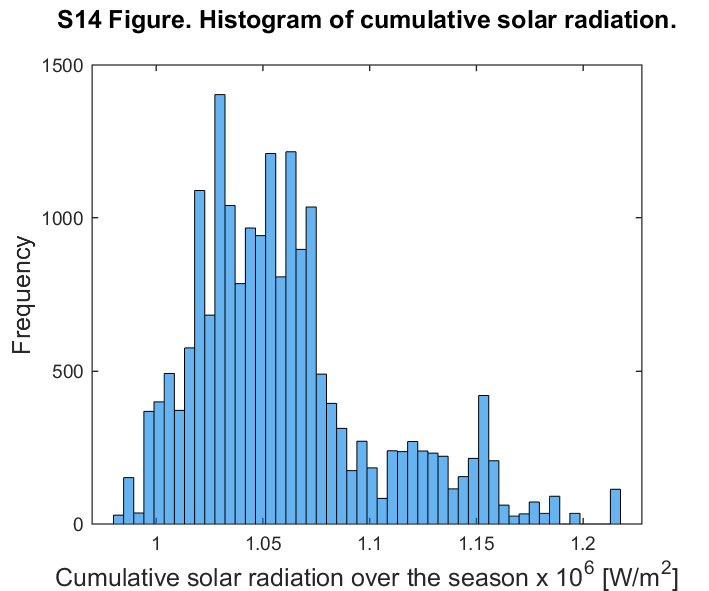

Supplement: S14 Fig — (TIF) [file pone.0184198.s014.tif]

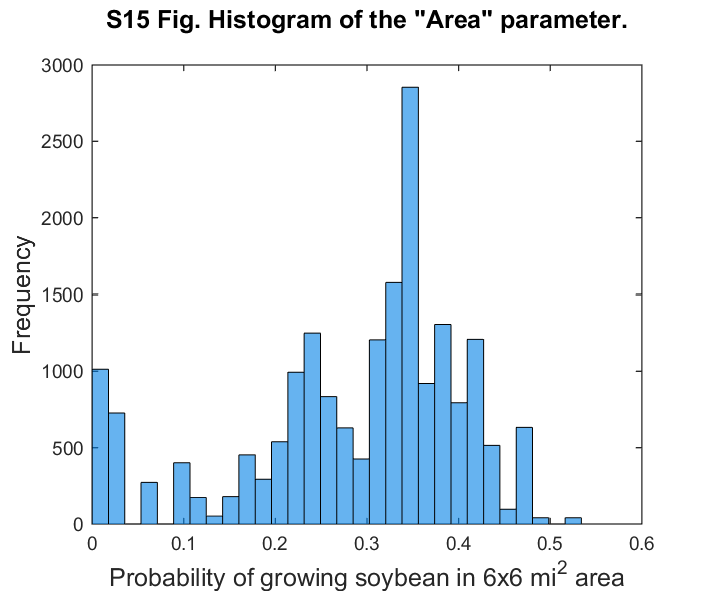

Supplement: S15 Fig — (TIF) [file pone.0184198.s015.tif]

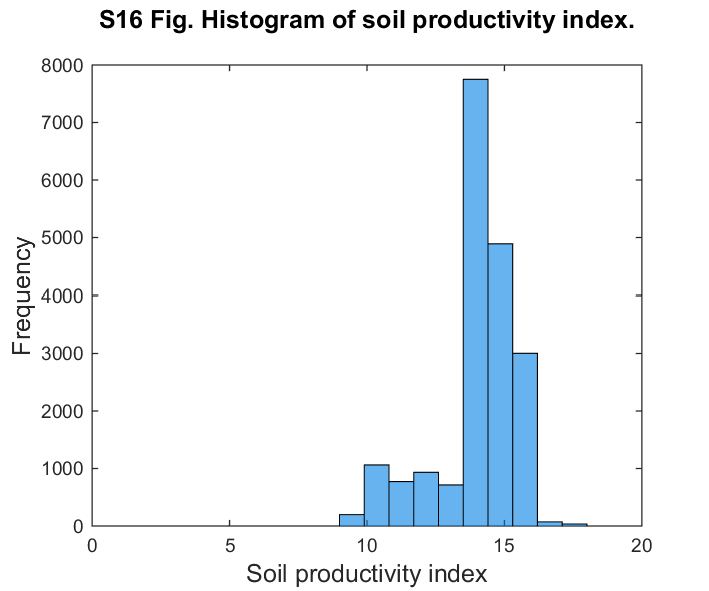

Supplement: S16 Fig — (TIF) [file pone.0184198.s016.tif]

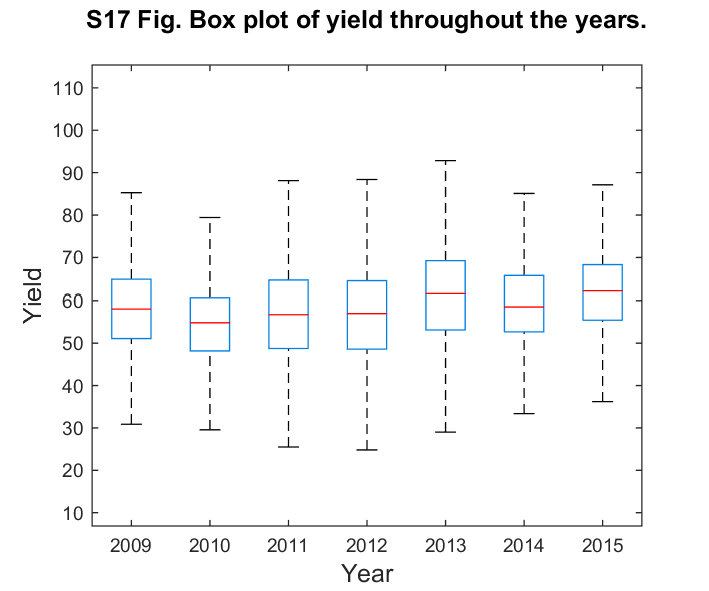

Supplement: S17 Fig — (TIF) [file pone.0184198.s017.tif]

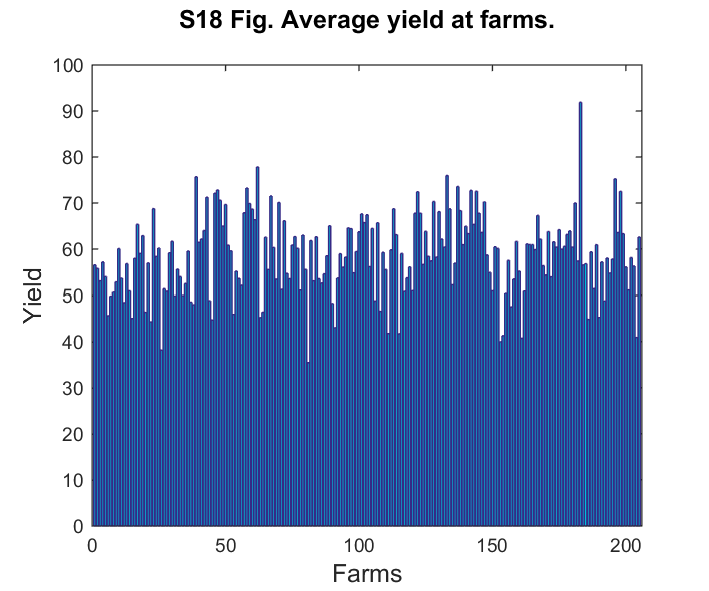

Supplement: S18 Fig — Yield was averaged across years and varieties. (TIF) [file pone.0184198.s018.tif]

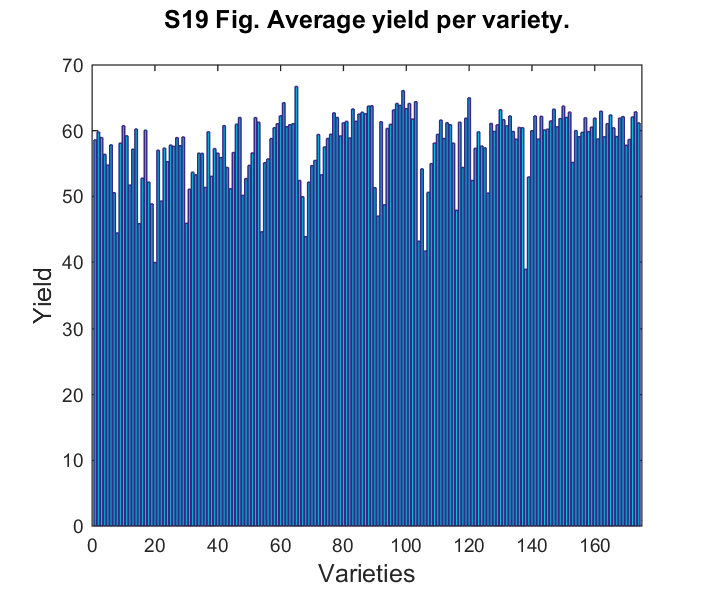

Supplement: S19 Fig — Yield was averaged across years and farms. (TIF) [file pone.0184198.s019.tif]
